# Promoting Resilience in Stress Management for Adolescents With Type 1 Diabetes: A Randomized Clinical Trial

**DOI:** 10.1001/jamanetworkopen.2024.28287

**Published:** 2024-08-19

**Authors:** Joyce P. Yi-Frazier, Marisa E. Hilliard, Maeve B. O’Donnell, Chuan Zhou, Britney M. Ellisor, Samantha Garcia Perez, Brenda Duran, Yuliana Rojas, Faisal S. Malik, Daniel J. DeSalvo, Catherine Pihoker, Miranda C. Bradford, Samantha Scott, Sridevi Devaraj, Abby R. Rosenberg

**Affiliations:** 1 Center for Clinical & Translational Research, Seattle Children’s Research Institute, Seattle, Washington; 2 Department of Psychosocial Oncology & Palliative Care, Dana-Farber Cancer Institute, Boston, Massachusetts; 3 Department of Pediatrics, Baylor College of Medicine and Texas Children’s Hospital, Houston; 4 Cambia Palliative Care Center of Excellence, University of Washington School of Medicine, Seattle; 5 Center for Child Health, Behavior, and Development, Seattle Children’s Research Institute, Seattle, Washington; 6 Division of General Pediatrics, Department of Pediatrics, University of Washington School of Medicine, Seattle; 7 Division of Diabetes/Endocrinology, Department of Pediatrics, University of Washington School of Medicine, Seattle; 8 Core for Biostatistics, Epidemiology and Analytics for Research (BEAR), Seattle Children’s Research Institute, Seattle, Washington; 9 Department of Psychology, University of Denver, Denver, Colorado; 10 Department of Pediatrics, Boston Children’s Hospital, Boston, Massachusetts; 11 Department of Pediatrics, Harvard Medical School, Boston, Massachusetts

## Abstract

**Importance:**

Type 1 diabetes (T1D) requires demanding self-management health behaviors, and adolescents with T1D are at risk for poor psychosocial and medical outcomes. Developing resilience skills may help adolescents with T1D and elevated distress navigate common stressors and achieve positive outcomes.

**Objective:**

To test the efficacy of the Promoting Resilience in Stress Management (PRISM) intervention on levels of hemoglobin A_1c_ (HbA_1c_), diabetes distress, self-management behaviors, resilience, and quality of life among adolescents.

**Design, Setting, and Participants:**

This phase 3, parallel, 1:1 randomized clinical trial that followed up 172 participants for 12 months was conducted from January 1, 2020, to November 30, 2022, at each of 2 children’s hospitals, in Seattle, Washington, and Houston, Texas. Participants were ages 13 to 18 years with T1D for at least 12 months and elevated diabetes distress.

**Intervention:**

PRISM, a manualized, skills-based, individual intervention program that teaches stress management, goal setting, reframing, and meaning-making, facilitated by a coach and accompanied by a digital app, was delivered in three 30- to 60-minute sessions approximately 2 weeks apart.

**Main Outcomes and Measures:**

The 2 primary outcomes, diabetes distress and HbA_1c_ levels, and 3 secondary outcomes, resilience, quality of life, and engagement in self-management behaviors, were assessed at baseline and 6 and 12 months after baseline. Linear mixed-effects regression models were used to evaluate associations between PRISM or usual care (UC) and these outcomes at both time points for the intention-to-treat population.

**Results:**

Among 172 adolescents (mean [SD] age, 15.7 [1.6] years), 96 were female (56%), and their baseline mean (SD) HbA_1c_ level was 8.7% (2.0%). No differences were evident between PRISM and UC recipients in HbA_1c_ levels (β, −0.21 [95% CI, −0.65 to 0.22]; *P* = .33) or diabetes distress (β, −2.71 [95% CI, −6.31 to 0.90]; *P* = .14) or any participant-reported outcome (eg, β, 2.25 [95% CI, −0.30 to 4.80]; *P* = .08 for self-management behaviors) at 6 months. At 12 months, there was no statistically significant difference between arms in HbA_1c_ levels (β, −0.26 [95% CI, −0.72 to 0.19]; *P* = .25); however, PRISM recipients reported significantly greater amelioration of diabetes distress (β, −4.59 [95% CI, −8.25 to −0.94]; *P* = .01) and improvement in self-management behaviors (β, 3.4 [95% CI, 0.9 to 5.9]; *P* = .01) compared with UC recipients.

**Conclusions and Relevance:**

The findings in this randomized clinical trial of psychosocial and behavioral improvements associated with PRISM at 12 months illustrate the value of a strengths-based intervention. Integrating resilience skills-building with traditional diabetes care may be a promising approach for improving outcomes among adolescents with T1D and elevated diabetes distress.

**Trial Registration:**

ClinicalTrials.gov number: NCT03847194

## Key Points

**Question:**

Is Promoting Resilience in Stress Management (PRISM), a psychosocial intervention designed to teach and develop skills to encourage resilience to stress, efficacious at improving hemoglobin A_1c_ (HbA_1c_), diabetes distress, and other patient-reported outcomes among adolescents with type 1 diabetes (T1D) and elevated diabetes distress?

**Findings:**

In this randomized clinical trial that included 172 adolescents with T1D and elevated diabetes distress, PRISM recipients had no change in HbA_1c_ levels but had significant improvements in diabetes distress and self-management behaviors 12 months after baseline measures.

**Meaning:**

These findings indicate that this resilience-building program is a promising approach for improving psychosocial and behavioral outcomes among adolescents with T1D and elevated diabetes distress.

## Teaser

This phase 3 randomized clinical trial assesses whether the intervention Promoting Resilience in Stress Management affects levels of hemoglobin A_1c_, diabetes distress, self-management behaviors, resilience, and quality of life for adolescents with type 1 diabetes and elevated diabetes distress.

## Introduction

For youths with type 1 diabetes (T1D), multiple management demands are needed to meet glycemic targets and reduce risk of acute and long-term diabetes-related complications.^1^ Over one-third of adolescents with T1D experience elevated diabetes distress, the emotional response associated with the burden of living with and managing this disease.^2,3^ Diabetes distress is associated with poor engagement in self-management behaviors, higher hemoglobin A_1c_ (HbA_1c_) levels, and higher risk of later medical comorbidities.^2,4^

The diabetes resilience model suggests that enhancing their resources may help adolescents manage stressors and achieve positive outcomes.^5^ Resilience resources—skills and behaviors that facilitate resilience to stress, such as stress management, goal setting, cognitive reframing, and meaning-making—have consistently been associated with positive behavioral and glycemic outcomes.^6,7,8^ Helping adolescents with T1D learn and apply individual resilience resources may improve psychosocial, behavioral, and glycemic outcomes.^9,10^

Based on the stress and coping theory,^11^ members of our team previously developed and demonstrated the feasibility and acceptability of Promoting Resilience in Stress Management (PRISM), a brief, skills-based psychosocial intervention targeting resilience resources among adolescents with medical stressors.^12,13,14^ PRISM has demonstrated efficacy in increasing resilience and decreasing distress among adolescents and young adults with cancer.^12^ In the present study, we aimed to evaluate the efficacy of PRISM to ameliorate diabetes distress and to improve HbA_1c_ levels, participant-reported resilience, quality of life, and self-reported management behaviors among adolescents with T1D with elevated diabetes distress. We hypothesized that PRISM would improve outcomes at 6 and 12 months after enrollment compared with usual care (UC).

## Methods

### Participants

This multisite, phase 3, parallel, 1:1 randomized clinical trial^15^ was conducted at Seattle Children’s Hospital in Seattle, Washington, and Texas Children’s Hospital in Houston from January 1, 2020, to November 30, 2022. The trial was approved by the Seattle Children’s Hospital institutional review board with a reliance agreement for study activities at Texas Children’s Hospital. The protocol is available in Supplement 1. Eligible participants were 13 to 18 years of age, fluent in English, diagnosed as having T1D for at least 12 months, had at least moderately elevated diabetes distress (assessed with Problem Areas in Diabetes-Teen [PAID-T^16^] scores ≥30) for 12 months or less prior to study enrollment, cognitively able to complete PRISM sessions and questionnaires, and provided written informed consent. Exclusion criteria included individuals who were wards of state, had severe comorbidities that significantly impacted daily management demands or health outcomes, or had prior PRISM participation. This study followed the Consolidated Standards of Reporting Trials (CONSORT) guideline for randomized clinical trials.^17^

### Recruitment and Randomization

A full description of eligibility screening and contacting of potential participants was previously described^15^ and is provided in Supplement 1. Private meetings between participants and research staff to discuss the consent form were conducted virtually beginning in April 2020 (4 participants were enrolled in person prior to this time).

Trial eligibility was confirmed through screening for moderately elevated diabetes distress via the PAID-T, a 14-item questionnaire assessing the perceived emotional burden of living with diabetes (Cronbach α, 0.86).^16^ While scores of 44 or higher indicate elevated distress,^16^ a score of 30 or higher was used to include individuals with moderate or greater distress. The PAID-T screening was conducted during routine diabetes care appointments or by study staff. Screening scores were used as baseline values unless obtained from routine clinical care more than 1 month prior to enrollment. In those cases, participants completed another PAID-T during enrollment.

After completing baseline data collection, study staff randomly assigned participants in REDCap 1:1 to the UC or PRISM group. The study statistician (M.C.B.) constructed the randomization algorithm with permuted blocks of various sizes, stratified by site.

### Procedures

Participants in both study arms received REDCap links to complete questionnaires every 3 months through 12 months after baseline. Questionnaires were considered missing if they were not received within 6 weeks of the due date. Participants received funds via a reloadable debit card for survey completion at each time point. The monetary value varied by time point to coincide with the length of the surveys: baseline ($20), 3 months ($10), 6 months ($30), 9 months ($10), and 12 months ($30). We obtained HbA_1c_ levels at baseline and 6 and 12 months after baseline.

### PRISM Program

#### Content

The PRISM intervention targets 4 resilience resources: stress management, goal setting, cognitive reframing, and meaning-making.^12,15^ PRISM was delivered in two 45- to 90-minute sessions plus one 30-minute summary session. The first session targeted stress management (deep breathing, relaxation, visualization, and mindfulness) and goal setting (how to identify and carry out realistic, actionable goals). The second session targeted cognitive reframing (recognizing, challenging, and replacing automatic negative thoughts with neutral or positive perceptions) and meaning-making (reflections and gratitude). The third, summary session invited participants to share PRISM skills with their parents (or parent) through review of the skills learned, reflection of their current strategies used to manage stressful times, and discussion of how their parents could support them. Although PRISM is disease agnostic,^18^ this program included diabetes-specific examples of skills to enhance the relevance to participants’ daily diabetes-related stressors. Sessions occurred via telehealth platforms that were compliant with the Health Insurance Portability and Accountability Act, telephone, or in person approximately every 2 weeks. Between sessions, participants had the option to use worksheets or the PRISM digital app for practice and tracking. PRISM coaches provided opportunities for brief telephone booster check-in calls every 2 weeks until 3 months after enrollment and monthly from months 3 through 6. Although no additional contact was given by PRISM coaches after 6 months, use of the PRISM app remained available.

#### Delivery and Fidelity

PRISM was administered by trained lay interventionists (PRISM coaches) with a bachelor’s degree or higher who were trained and supervised by a PhD-level study supervisor. Standardized PRISM training protocols have been previously described.^12,13^ All PRISM sessions were audio recorded for fidelity monitoring. For each PRISM coach, the first 3 sessions and 1 of any 6 randomly selected sessions were scored for fidelity with a standardized content review tool. If fidelity was not met, remediation included coach retraining.

### Usual Care

All participants received usual health care for T1D. At both study sites, this aligned with the American Diabetes Association standard of care^19^ and involved a multidisciplinary team of diabetes specialists, including a primary clinician, diabetes educators, nutritionists, and social workers. Both study sites have systems in place to make referrals for mental or behavioral health care when clinically indicated. Participants in the usual care (UC) arm received no additional intervention during the study period and were given access to the PRISM mobile app following completion of the final 12-month data collection.

### Study Outcomes

The primary aim of the study was to examine the efficacy of the PRISM intervention on the main outcomes: HbA_1c_ levels and diabetes distress 6 months after the baseline. Secondary outcomes included participant-reported resilience, quality of life, and engagement in self-management behaviors at 6 and 12 months.

### Study Instruments

#### Clinical Variables

The HbA_1c_ levels were collected at baseline and 6 and 12 months after the baseline. If available from a routine diabetes care appointment within specified windows (within 4 weeks), point of care values were used. Due to an increase in telehealth appointments during the COVID-19 pandemic, dried blood spot collection for HbA_1c_ was also available (details previously published).^15^

Other clinical variables, including insulin delivery method (ie, multiple daily injections or insulin pump), use of continuous glucose monitoring, and comorbidities (eg, celiac disease, thyroid disease, attention-deficit/hyperactivity disorder, depression, and anxiety) were collected via electronic health record abstraction.

#### Participant-Reported Outcomes

In addition to the PAID-T^16^ measure, participants completed the following validated questionnaires at baseline and 6 and 12 months after baseline. Diabetes distress and both measures of resilience described below were also assessed at 3 and 9 months in an abbreviated survey. The 10-item Connor-Davidson Resilience Scale (CD-RISC)^20,21^ captures the perceived ability to cope with adversity and has been used with adolescents (Cronbach α = 0.81).^21^ Higher scores indicate greater perceived resilience.

##### Diabetes Resilience

The Diabetes Strengths and Resilience (DSTAR) measure^3,22^ is a 12-item measure of adolescents’ perceptions of diabetes-related strengths, including confidence to manage diabetes and ability to access diabetes-related support (Cronbach α = 0.73). Higher scores indicate greater strengths.

##### Diabetes Quality of Life

Age-specific versions of the Type 1 Diabetes and Life (T1DAL) measure^23,24^ include the T1DAL-Teen (23 items) for ages 12 through 17 (Cronbach α = 0.85) and T1DAL-Young Adult (27 items) for age 18 years (Cronbach α = 0.78). Respondents rate positive and negative aspects of their life with T1D. All T1DAL versions calculate total scores on a 100-point scale. Higher scores indicate better quality of life.

##### Engagement in Self-Management Behaviors

The Diabetes Self-Management Questionnaire (DSMQ)^25^ is a validated 9-item questionnaire assessing self-reported engagement in T1D self-management tasks (Cronbach α = 0.67). This questionnaire has been used with adolescents, and higher scores indicate higher engagement.

##### Demographic and Family Variables

Youth self-reported race and ethnicity (aligned with the National Institutes of Health reporting categories such that participants could check any of the following applicable options: American Indian or Alaska Native; Asian; Black or African American; Native Hawaiian or Other Pacific Islander; White; and other, with an open text box), and ethnicity (Hispanic or Latino: yes or no). We included race and ethnicity as standard covariates in our analyses. For gender, participants were given options of male, female, or other, with an open text box. Insurance plans (public, private, and other) and any missing demographic responses were abstracted from the electronic health record.

### Statistical Analysis

In similar populations of youths with T1D and elevated distress (mean [SD] PAID-T scores, 49.3 [16.2]), reported mean (SD) HbA_1c_ values were 9.0% (1.9%).^4,16^ A sample size of 120 participants would achieve 80% power to detect a minimum difference of 1.1% in postintervention mean HbA_1c_ levels and a minimum difference of 8.4 in mean PAID-T scores, based on 2-sample independent *t* tests. Assuming 20% attrition, we planned to recruit 160 adolescents (80 in each arm), for an evaluable sample size of 120 (60 in each arm).

We conducted intention-to-treat analyses with all randomized participants. Baseline demographics and clinical characteristics were summarized using descriptive statistics and compared between treatment arms using χ^2^ or Wilcoxon rank sum tests. The HbA_1c_ value and patient-reported outcomes (PROs) were treated as continuous variables. For primary analyses, we applied covariate-adjusted linear mixed-effects regression models^26^ to the repeated assessment outcomes to test the hypothesis that adolescents randomized to receive PRISM would demonstrate greater improvement than adolescents randomized to receive UC for all outcomes. Effect sizes for the continuous outcomes were measured using the Cohen *d*.^27^

The mixed-effects regression models included time as a discrete predictor, intervention group, group-by-time interaction, and individual-specific random effects to account for within-individual correlations due to repeated assessments. The study site was the stratification factor and was controlled for in all the models. The parameter of interest was the group-by-time interaction, which estimated the difference between treatment groups in changes over time for a given outcome. Statistical significance was assessed based on the *F* test with the Kenward-Roger method for degrees of freedom approximation.^28^

We checked missing data rates and patterns and performed sensitivity analyses for linear mixed-effects regression models on multiple imputed datasets using multivariate imputation with chained equations.^29^ All statistical analyses were conducted using R statistical software, version 4.3.1, and related packages (R Project for Statistical Computing).^30^ A 2-sided *P* < .05 was considered statistically significant.

## Results

### Study Population

**Figure 1.**
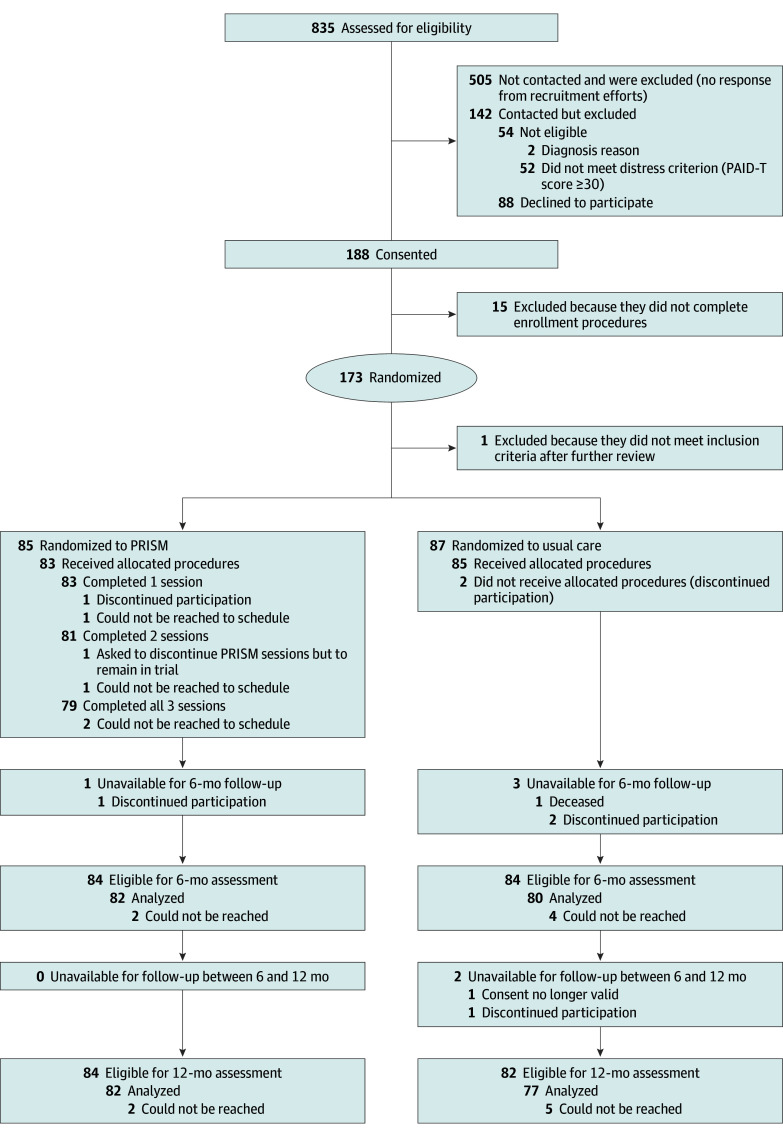
CONSORT Diagram of the Promoting Resilience in Stress Management (PRISM) Randomized Clinical Trial Through 12 Months of Follow-Up

Of 835 potential participants prescreened for eligibility, we reached 330 to assess study participation. Of those, 52 did not meet the PAID-T screening criterion, and 2 did not meet other eligibility criteria. Of the 276 confirmed eligible, 88 declined to participate (Figure 1). Thus, 188 provided informed consent (68%); of these, 173 (92%) completed all enrollment procedures and were randomized. One participant was deemed ineligible immediately following randomization based on follow-up clarifications regarding diabetes type, leaving 85 randomized to the PRISM and 87 randomized to the UC groups.

Of 172 participants who provided informed consent and were randomized, the mean (SD) age was 15.7 (1.6) years, and the mean (SD) HbA_1c_ was 8.7% (2.1%) (to convert to proportion of total hemoglobin, multiply by 0.01). Self-reported gender included 96 female (56%), 68 male (40%), and 8 other (5%) participants. In total, 26 participants identified their race as African American or Black (15%), 11 as American Indian or Alaska Native or Asian (6%), 125 as White (73%), and 10 as other or multiple races (6%). No participants identified as Native Hawaiian or Other Pacific Islander. Hispanic ethnicity was endorsed by 33 participants (19%). Demographic and medical characteristics are reported in Table 1, and baseline PROs, in eTable 1 in Supplement 2.

**Table 1.** Participant Demographic and Clinical Characteristics

Characteristic	Participants, No. (%)		
	Overall (n = 172)	Usual care (n = 87)	PRISM (n = 85)
Age, mean (SD), y	15.7 (1.6)	15.7 (1.6)	15.7 (1.6)
Gender			
Female	96 (56)	52 (60)	44 (52)
Male	68 (40)	33 (38)	35 (41)
Other	8 (5)	2 (2)	6 (7)
Race^a^			
African American or Black	26 (15)	15 (17)	11 (13)
American Indian or Alaska Native	4 (2)	2 (2)	2 (2)
Asian	7 (4)	4 (5)	3 (4)
White	125 (73)	62 (71)	63 (74)
Other or multiple races	10 (6)	4 (5)	6 (7)
Ethnicity			
Hispanic	33 (19)	11 (13)	22 (26)
Non-Hispanic	139 (81)	76 (87)	63 (74)
Insurance			
Private	127 (75)	69 (80)	58 (69)
Public	42 (25)	17 (20)	25 (30)
Other	1 (1)	0 (0)	1 (1)
HbA_1c_, mean (SD), %	8.7 (2.1)	8.6 (2.2)	8.8 (1.9)
Type 1 diabetes duration, mean (SD), y	6.7 (4.0)	6.4 (4.1)	7.1 (4.0)
Continuous glucose monitor use	124 (72)	63 (72)	61 (72)
Insulin pump use	115 (67)	60 (69)	55 (65)
Comorbidity			
Celiac disease	10 (6)	4 (5)	6 (7)
Thyroid disease	22 (13)	12 (14)	10 (12)
ADHD	18 (10)	7 (8)	11 (13)
Depression	23 (13)	13 (15)	10 (12)
Anxiety	22 (13)	12 (14)	10 (12)
Other	45 (26)	19 (22)	26 (31)

Abbreviations: ADHD, attention-deficit/hyperactivity disorder; HbA_1c_, hemoglobin A_1c_; PRISM, Promoting Resilience in Stress Management.

SI conversion factor: To convert HbA_1c_ percentage to proportion of total, multiply by 0.01.

^a^ Youth self-reported race and ethnicity aligned with the National Institutes of Health reporting categories, such that participants could check any of the following applicable options: American Indian or Alaska Native; Asian; Black or African American; Native Hawaiian or Other Pacific Islander; White; and other, with an open text box; no participants identified as Native Hawaiian or Other Pacific Islander.

### Retention and Missing Values

Study retention was high for both arms. Baseline HbA_1c_ value, family member other than mother providing diabetes care, and baseline diabetes resilience were associated with missing data at follow-up assessments.

At 6-month, 1 PRISM participant discontinued participation in the trial (Figure 1). Among 84 remaining eligible participants for the 6-month assessment, 2 could not be reached for data collection. In total, 75 participants completed both HbA_1c_ testing and questionnaires, 5 missed HbA_1c_ testing but completed questionnaires, and 2 completed HbA_1c_ testing but not questionnaires. For the UC participants at 6-month, 1 died due to a T1D-unrelated reason, and 2 discontinued participation. Among 84 eligible UC participants, 4 could not be reached for data collection. In total, 71 participants completed both HbA_1c_ testing and questionnaires, 3 missed HbA_1c_ testing but completed questionnaires, and 6 completed HbA_1c_ testing but not questionnaires.

By 12 months, no additional PRISM participants were unavailable for follow-up, leaving 84 remaining eligible for 12-month assessment (Figure 1). Of these participants, 2 could not be reached for 12-month data collection, 68 completed both HbA_1c_ testing and questionnaires, 9 missed HbA_1c_ testing but completed questionnaires, and 5 completed HbA_1c_ testing but not questionnaires. For UC, 1 person discontinued participation in the trial, and 1 person had an invalid consent due to a family situation, leaving 82 participants eligible for the 12-month assessment (Figure 1). Of these participants, 5 could not be reached for data collection, 59 completed both HbA_1c_ testing and questionnaires, 12 missed HbA_1c_ testing but completed questionnaires, and 6 completed HbA_1c_ testing but not questionnaires.

### Intervention Engagement and Fidelity

Of 85 participants randomized to receive PRISM, 83 (98%) completed at least 1 session, 81 (95%) completed sessions 1 and 2, and 79 (93%) completed all 3 sessions (Figure 1). Of those participants who did not complete the first 2 PRISM sessions, 2 could not be reached to schedule, 1 chose to forgo PRISM sessions, and 1 withdrew consent due to school or time demands. The 2 additional participants who did not complete the third session could not be reached. The PRISM app, which was an optional component for tracking and practice, was used by 48 of 85 participants.

A total of 243 video sessions were conducted. Fidelity was endorsed in 100% of the fidelity checks.

### Primary Outcomes: HbA_1c_ and Diabetes Distress

At 6 months, adjusted analyses detected no statistically significant differences between PRISM and UC for HbA_1c_ values (β, −0.21 [95% CI, −0.65 to 0.22]; *P* = .33) or diabetes distress (β, −2.71 [95% CI, −6.31 to 0.90]; *P* = .14) (Table 2). Absolute effect sizes were 0.08 (95% CI, −0.24 to 0.39) (negligible) for HbA_1c_ values and 0.27 (95% CI, −0.59 to 0.05) (small) for diabetes distress (eTable 2 in Supplement 2).

**Table 2.** Adjusted Results for Outcomes of PRISM vs Usual Care Over 12 Study Months^a^

Outcome	6-mo Assessment		12-mo Assessment	
	β (95 CI)^b^	*P* value^c^	β (95 CI)	*P* value^c^
Primary				
HbA_1c_	−0.21 (−0.65 to 0.22)	.33	−0.26 (−0.72 to 0.19)	.25
Diabetes distress (PAID-T)	−2.71 (−6.31 to 0.90)	.14	−4.59 (−8.25 to −0.94)	.01
Secondary				
Resilience (CD-RISC)	−0.36 (−2.17 to 1.45)	.70	−0.12 (−1.95 to 1.72)	.90
Diabetes Resilience (DSTAR)	0.97 (−0.95 to 2.89)	.32	1.83 (−0.11 to 3.77)	.07
Self-management behaviors (DSMQ)	2.25 (−0.30 to 4.80)	.08	3.22 (0.67 to 5.76)	.01
Health-related quality of life (T1DAL)	0.63 (−3.01 to 4.26)	.74	3.53 (−0.15 to 7.21)	.06

Abbreviations: CD-RISC, Connor-Davidson Resilience Scale; DSMQ, Diabetes Self-Management Questionnaire; DSTAR, Diabetes Strengths and Resilience; HbA_1c_, hemoglobin A_1c_; PAID-T, Problem Areas in Diabetes-Teen; PRISM, Promoting Resilience in Stress Management; T1DAL, Type 1 Diabetes and Life.

^a^ Based on linear mixed-effects regression models, with study group and time point main effects, and a group-by-time interaction. The stratification factor study site was controlled as a covariate in all the models.

^b^ β corresponds to the group-by-time interaction in the regression model.

^c^ Based on the *F* test with the Kenward-Roger approximation to estimate the degrees of freedom.

**Figure 2.**
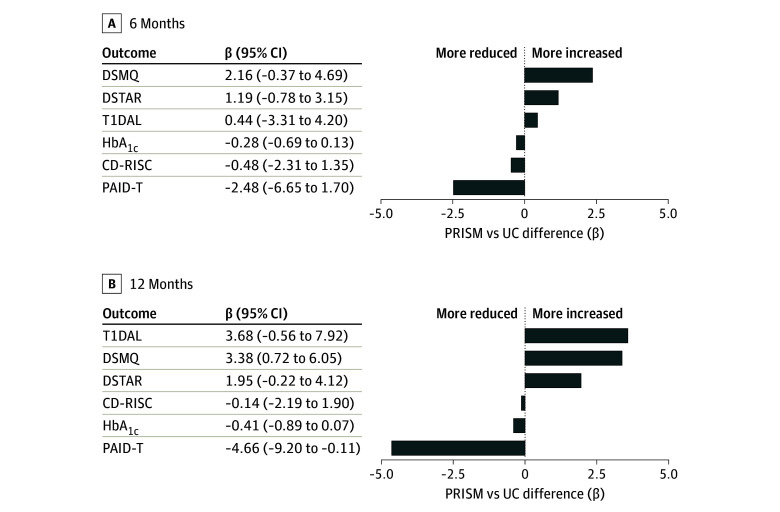
Promoting Resilience in Stress Management (PRISM) vs Usual Care (UC) Differences in Outcomes 6 and 12 Months After Baseline CD-RISC represents the Connor-Davidson Resilience Scale (resilience); DSMQ, Diabetes Self-Management Questionnaire (adherence); DSTAR, Diabetes Strength and Resilience measure (diabetes resilience); HbA_1c_, hemoglobin A_1c;_ PAID-T, Problem Areas in Diabetes-Teen version (diabetes distress); and T1DAL, Type 1 Diabetes and Life (diabetes-specific health-related quality of life).

At 12 months, there was no significant difference in HbA_1c_ values between arms (β, −0.26 [95% CI, −0.72 to 0.19]; *P* = .25). PRISM was associated with more improvement than UC in diabetes distress (β, −4.59 [95% CI, −8.25 to −0.94]; *P* = .01 using PAID-T) (Figure 2). Absolute effect sizes were 0.11 (95% CI, −0.23 to 0.44) for HbA_1c_ (negligible) and 0.37 (95% CI, −0.70 to −0.05) (small to medium) for diabetes distress (eTable 2 in Supplement 2).

### Secondary Outcomes: PROs

At 6 months, there were no significant differences for general resilience, diabetes strengths, diabetes-related quality of life, or self-management behaviors (eg, β, 2.25 [95% CI, −0.30 to 4.80]; *P* = .08) (Table 2). Absolute effect sizes ranged from 0.07 (negligible) to 0.26 (small) (eTable 2 in Supplement 2).

At 12 months, PRISM was associated with more improvement than UC in diabetes self-management behaviors (β, 3.22 [95% CI, 0.67-5.76; *P* = .01 using the DSMQ) (Figure 2). The absolute effect size was 0.33 (95% CI, 0.03-0.63) (small to moderate). Results for diabetes resilience (β, 1.83 [95% CI, −0.11 to 3.77]; *P* = .07) and health-related quality of life (β, 3.53 [95% CI, −0.15 to 7.21]; *P* = .06) had absolute effect sizes of 0.35 (95% CI, 0.03-0.68) for diabetes resilience and 0.39 (95% CI, 0.06-0.72) for health-related quality of life (both small to moderate). No significant differences were detected for general resilience, with a negligible absolute effect size of 0.03 (95% CI, −0.36 to 0.29) between groups.

### Sensitivity Analysis

Sensitivity analyses were conducted under the missing-at-random assumption. Sensitivity analyses with multiple imputations demonstrated that all findings remained the same as those for the main analyses (eTable 3 in Supplement 2).

## Discussion

In this phase 3, multisite randomized clinical trial involving adolescents with elevated diabetes distress, PRISM did not impact HbA_1c_ levels compared with UC; however, there was some promise for PRISM to reduce diabetes distress and affect T1D self-management behaviors 12 months after enrollment. These findings represent the first, to our knowledge, extension of the efficacy of PRISM beyond a pediatric cancer population, highlighting the potential psychosocial and behavioral benefits of this resilience promotion intervention across complex health conditions. Given the known risks for this population, strengthening resilience resources to effectively manage T1D-related challenges may be an important clinical strategy.

Although diabetes distress was ameliorated among participants in both study arms over time, PRISM recipients achieved larger improvements compared with UC recipients. The evidence for the effect of PRISM at 12 months, but not at 6 months, countered our hypothesis, which was based on previous work in similarly aged youths with cancer.^12^ The delayed effect of PRISM in improving outcomes in adolescents with T1D may reflect at least 3 factors: (1) The acuity of cancer-related stressors may explain earlier benefits from PRISM; adolescents with T1D have lived with their disease longer and have more sustained stressors. For them, additional practice and time may be needed for an effect. (2) Given the eligibility requirement of elevated diabetes distress in this trial, participants may have experienced a more sustained response to stress that needed additional time to change. Prospective, longitudinal data from members of our team suggest that earlier intervention or longer-term follow-up may be needed.^31^ (3) Conducting a psychosocial intervention study during the early stages of the COVID-19 pandemic made it difficult to discern the expected level and acuity of stress. Across our 2 study sites, there was wide variability in lockdown timelines, availability of vaccines, statewide mandates, and community exposure rates. These factors may have affected health behaviors and the timing and degree of the intervention’s impact. Thus, as the effects of psychosocial interventions in T1D are not typically long-lasting,^32,33^ the 12-month outcome data from this present trial represent the promising effect of PRISM for this population.

Despite the challenges of COVID-19, enrollment and retention rates were high. After people adapted to the fully remote procedures necessitated by the pandemic,^34^ 12-month completion rates higher than 90% bolstered the generalizability of the findings, even in the context of the pandemic-related adaptations.^34,35^ The finding that more UC recipients were unavailable for follow-up was similar to other PRISM trials^12^ and may reflect a perceived lack of benefit for participants who did not receive the intervention. For PRISM recipients, in addition to high retention, high intervention engagement rates were consistent with other PRISM trials.^12,13,14^ The finding that 95% of participants completed the 2 core sessions supports the feasibility, acceptability, and perceived value of this program.

For self-management behaviors, UC recipient scores declined while PRISM recipients maintained engagement. The null effect of PRISM on glycemic control is consistent with other behavioral studies showing improvements in behavioral outcomes but not in HbA_1c_ levels.^36^ Self-management behaviors fluctuate during adolescence, and stress (including from a global pandemic) can undermine self-management.^37,38,39,40^ Thus, our results support addressing stress as a means to improving self-management behaviors and, ultimately, HbA_1c_ levels.

### Limitations

The limitations of this study include challenges to generalizability. The sample included only English-speaking youths, and White and non-Hispanic participants were overrepresented. However, sensitivity analyses did not suggest differences in outcomes across racial or ethnic groups. Progress is being made to translate PRISM into other languages. We were unable to reach many potential participants, reducing representation of people who may have experienced more challenges or barriers to engagement during the pandemic. The pandemic may have impacted participants differently across the study period, particularly given distinct pandemic precautions and timelines in Washington and Texas. Finally, alternative comparators (beyond UC) may be useful in future research.^41^

## Conclusions

In this randomized clinical trial of adolescents with T1D and elevated diabetes distress, PRISM recipients had no change in HbA_1c_ levels but had significant improvements in diabetes distress and self-management behaviors 12 months after baseline measures. These findings suggest that future research should further study resilience skills building for adolescents with T1D and diabetes distress, such as identifying optimal timing and dose to bolster the 12-month effects observed in this trial and longer-term follow-up periods to study sustained improvements and potential later effects on HbA_1c_ values. Examination of a potential prevention effect of PRISM through the targeting of individuals with lower levels of diabetes distress may be another meaningful approach to impacting outcomes. Overall, these findings suggest that the strengths-based PRISM intervention represents a feasible and moderately efficacious approach to promote psychosocial and behavioral health benefits among adolescents with T1D and elevated diabetes distress.

## Notes

Supplement 1.
Trial Protocol

Supplement 2. **eTable 1.** Unadjusted Results for Outcomes of PRISM vs Usual Care Over the 12 Study Months **eTable 2.** Effect Sizes on Outcomes at 6 and 12 Months **eTable 3.** Adjusted Regression Analysis With Multiple Imputations (MI)

Supplement 3.
Data Sharing Statement
