# Supplementary material for: Promoting Resilience in Stress Management for Adolescents With Type 1 Diabetes: A Randomized Clinical Trial
Source: JAMA Netw Open. 2024 Aug 19;7(8):e2428287. doi: 10.1001/jamanetworkopen.2024.28287 (PMC11333977; doi:10.1001/jamanetworkopen.2024.28287)
Supplement: Supplement 1. — Trial Protocol [file jamanetwopen-e2428287-s001.pdf]

PROJECT TITLE:

The Promoting Resilience in Stress Management (PRISM) Intervention: a multi-site randomized controlled trial for adolescents with type 1 diabetes

PRINCIPAL INVESTIGATOR:

Joyce Yi-Frazier, PhD
Seattle Children's Research Institute
1900 9th Ave, JMB 9-C
Seattle, WA
206-884-1456
joyce.yi-frazier @seattlechildrens.org

Coordinating Site: Seattle Children's Research Institute

Participating Site: Baylor College of Medicine/Texas Children's Hospital

Dr. Yi-Frazier assumes responsibility for the conduct of the study.

ClinicalTrials.gov Identifier: NCT03847194

Contents

1. Objectives 3
2. Background 4
3. Study Endpoints 7
4. Drugs, Devices and Biologics 8
5. Procedures Involved 8
6. Data and Biospecimen Banking 14
7. Sharing of Results 15
8. Study Timelines 15
9. Study Population 16
10. Number of Subjects 18
11. Withdrawal of Subjects 19
12. Risks to Subjects 20
13. Potential Benefits to Subjects 22
14. Data Analysis/Management 22
15. Confidentiality 24
16. Provisions to Monitor Data to Ensure the Safety of Subjects 25
17. Use of Social Media 27
18. Research Related Injury 27
19. Recruitment Methods 27
20. Consent/Assent Process 29
21. Process to Document Consent in Writing 34
22. HIPAA Authorization and RCW Criteria 35
23. Payments/Costs to Subjects 36
24. Setting 37
25. Resources Available 37
26. Coordinating Center Procedures 39
27. International Center for Harmonization of Good Clinical Practice (ICH-GCP) 40

1. Objectives

1.1. Purpose, specific aims, or objectives:

Adolescents with type 1 diabetes (T1D) are at high risk for elevated distress related to their diabetes.[1-3] This is of critical concern as high diabetes distress is strongly associated with poor glycemic control, adherence, and other psychological disorders.[1,4-11] Given that intensive treatment is required to adequately manage diabetes, adolescents with T1D face unique challenges at their developmental stage, which universally is a period of increased risk.[12,13] Integrated psychosocial care is suboptimal despite national and international recommendations[14,15] due to high costs, limited availability of proven interventions, and a lack of trained clinicians to deliver those interventions.[16]

One barrier to improving the experiences and outcomes for adolescents with T1D may be the lack of opportunities for them to develop "resilience resources," or skills to help manage challenges, such as stress-management, goal-setting, positive reframing, and meaning-making. Promoting resilience resources may mitigate negative outcomes such as diabetes distress, facilitate adherence, and help adolescents better navigate the challenges of T1D, particularly as they begin to become more independent from their caregivers in all facets of their life.[17-21]

Our research program is built on the premise that promoting resilience resources will reduce distress and improve outcomes. We have developed conceptual frameworks of resilience in pediatric illness,[20,22,23] affirmed associations between resilience resources and outcomes,[21,24-28] and developed and piloted a novel resilience resources intervention (Promoting Resilience in Stress Management, PRISM).[29] PRISM is a skills-based, one-on-one training program consisting of two, 45-60 minute sessions followed by a family-meeting and supported by booster sessions and a digital app designed specifically for the adolescent population. Feasibility has been tested in adolescents with diabetes,[29] cancer,[29] cystic fibrosis,[30] and currently in patients with craniofacial disorder and end-stage renal disease. We recently completed a pilot Randomized Controlled Trial (RCT) comparing PRISM to usual care among adolescents with cancer. Results suggest PRISM is feasible, highly acceptable, and associated with higher patient-reported resilience and quality of life, and lower risk of psychological distress 6 months following enrollment.[31] While initial findings for PRISM are promising in multiple groups, its efficacy among adolescents with T1D has not been tested.

Improving diabetes distress and health outcomes for adolescents with T1D requires innovative, developmentally-targeted, cost-feasible interventions designed to help patients effectively manage the stressors and challenges of T1D management in the context of everyday adolescent life. We propose a multi-site RCT comparing PRISM to usual care among youth with T1D (13-18 years with suboptimal glycemic control (elevated A1C) and diabetes distress as measured with a validated scale).

1.2. Hypotheses to be tested:

1. **Evaluate PRISM's efficacy in reducing A1C.** The primary outcome will be A1C at 6-months post-enrollment. ***Hypothesis:*** *PRISM will be associated with lower A1C compared to usual care at 6-months post-enrollment.* As an exploratory aim, for the subset of participants on continuous glucose monitors (approx. 30% of expected population), we will also examine PRISM's effect on sensor glucose time in range. A1C and time in range will be assessed for a total duration of 12 months post-intervention.
2. **Evaluate PRISM's efficacy in improving diabetes distress.** Self-reported adherence and other Patient-Reported Outcomes (PROs), including resilience and quality of life will also be

assessed by adolescents and their caregivers. ***Hypothesis:*** *PRISM will be associated with lower diabetes-related distress and depressive symptoms and higher resilience, quality of life,benefit-findingand self-reported adherence 6-months post-intervention compared to usual care*.

2. Background

2.1. Relevant prior experience and gaps in current knowledge:

Over a third of adolescents with T1D face elevated diabetes-specific distress.[1-3] Diabetes distress, or negative emotional response to the burden of diabetes, is concerning given its strong and established association with poor glycemic control.[1,9-11] **A potential explanation for elevated diabetes distress is that adolescents lack skills to manage their stressors**. Several personal resources and skills are consistently associated with resilience to stress, which may mitigate negative and facilitate positive outcomes.[28,32,33] These include stress-management, problem-solving and goal-setting skills, cognitive reframing, and the ability to make meaning from adversity.[17-21,34] We term this group of variables "resilience resources." Previous work in youth with T1D has shown resilience resources relate to glycemic control, diabetes distress, and other important outcomes;[17-21] hence, skills-based interventions bolstering resilience resources may improve both diabetes distress and A1C.[35-38]

The Promoting Resilience in Stress Management (**PRISM) Intervention has the potential to reduce distress in a feasible, age-appropriate intervention while improving health outcomes in adolescents with T1D.** PRISM was developed based on stress and coping theory to be a brief, disease non-specific, skills-based intervention targeting adolescent resilience resources (**Table 1**). PRISM teaches 4 pillars of resilience including stress management, goal setting, cognitive restructuring and benefit-finding in a one-on-one training program consisting of two, 45-60 minute sessions and supported by 6-months of booster sessions and a fully developed digital app for practice and tracking. A family session follows the individual sessions and was designed for the adolescent to communicate to his/her caregiver(s) the skills learned, and how the caregiver(s) could best support ongoing practice. Results from a pilot randomized controlled trial (RCT) in cancer suggest PRISM is associated with improved adolescent distress and resilience scores.[31] Despite differences in illness acuity, these themes are also relevant to adolescents with T1D and have been reported to be desirable in this population.[29]

**Table 1. PRISM intervention content**			
**Pillar***	**Details**	**Format (in-person, by phone, or video conference)**	**Stress and Coping Theory Constructs**
1. Managing Stress	Mindfulness techniques, relaxation strategies, breathing	1:1	Situational factors
2. Goal-setting	Setting SMART goals, planning for roadblocks	1:1	Situational factors
3. Positive Reframing	Recognizing and reframing negative self-talk	1:1	Coping processes
4. Meaning Making	Identifying gratitude or meaning from disease experience	1:1	Coping processes
"Coming Together"	Discussion about what was learned, what helped, what caregivers can do to help	Family meeting	Both
Boosters	Reflections on skills, opportunities for refreshers and further development of skills	1:1	Both
Skill Practice	Between-session exercises to practice, further develop and track skills	Digital App (or paper/pencil)	Both
**Pillars 1-2 and 3-4 delivered in 2 sessions approximately 2 weeks apart, "Coming Together" follows 2-weeks after.*			

2.2. Relevant preliminary data:[1]

Preliminary studies. Our central hypothesis is that promoting resilience resources will improve outcomes for adolescents with T1D. We define "resilience resources" as several modifiable factors of cognitive, emotional and physical well-being, including individual perceptions of stress, and abilities to set goals and find meaning from adversity.[39] We have followed a step-wise approach to investigate our hypothesis.

Concept development. First, we conducted cross-sectional, mixed-methods studies across diverse populations of chronic/serious illness to explore the construct of resilience in association with distress and health outcomes. **Our research group consistently confirmed associations between lower resilience resources, higher distress, and worse health outcomes.**[21,23,25,40] In adolescents with diabetes specifically, low resilience was associated with higher distress, lower quality of life, and poorer glycemic control.[21] Prospective, longitudinal studies established evidence for resilience resources buffering the effect of stress on worsening glycemic control, thereby serving as a protective factor.[25,26,28,41] Subsequent thematic analyses in adolescents with cancer suggested that adolescents endorse the need for strong resilience resources, but that they lack the skills.[22]

Intervention Development. These studies provided rationale for the design of a **novel intervention to promote resilience resources, "Promoting Resilience in Stress Management"** (PRISM, Table 1).[29] PRISM is based on stress and coping theory,[42] our prior research, and successful interventions described in other populations.[43-45] It is disease non-specific and manualized (i.e., it has been standardized via comprehensive protocols). The initial design was refined with expert opinion and interviews with adolescent patients, psychologists, and social workers. PRISM's overall objective is to increase resilience resources at times of high stress, thereby giving adolescents skills to alleviate distress, and improve health outcomes.

Feasibility and Acceptability of PRISM. We completed a formative study of PRISM among adolescents with T1D and cancer to determine the optimal content and timing of PRISM sessions.[29] **We found it to be feasible and highly valuable to adolescent patients and parents.** Eighty percent of participants completed the intervention and feedback was universally positive. One primary disease-specific difference emerged: participants with diabetes preferred fewer but longer sessions whereas those with cancer preferred more frequent, shorter sessions.

Thus, the four PRISM intervention pillars were combined into two sessions for the diabetes participants. We successfully trained 3 interventionists (all research associates (RAs) with undergraduate degrees). Each underwent at least 8 hours of training, and tracking of per-patient intervention delivery time suggested 4 hours of face-to-face time, 2 hours tracking/scheduling, and up to 2 hours of fidelity review (total ~8 hours per patient).

PRISM Pilot RCT (clinicaltrials.gov NCT 02340884). Next, in a pilot RCT of PRISM in adolescents and young adults (AYA) with cancer, we refined processes of enrollment, randomization, implementation, and data collection. We completed our target enrollment of 100 AYAs with cancer ahead of schedule, with a 78% enrollment rate. Final pilot results suggest **the intervention was associated with improved patient-reported resilience and distress** with moderate effect sizes.[31]

PRISM App Development. Finally, a digital app has been designed, developed and beta-tested **specifically for adolescents**, providing supplemented practice and resources throughout the intervention. The app was designed and built by two Seattle-based companies [Artefact (artefactgroup.com) and General UI (generalui.com)], prior PRISM recipients, and the SCH Digital Health team. The current platform is available for iOS and Android programs and includes the same practice opportunities (e.g., directed practice with goal-setting, journaling of gratitudes, etc.) as paper-pencil worksheets. The digital interface also allows study staff to track how frequently users open each practice page, how long they stay on it, and how they use it over time. The app is not available on the app store, so the study team can closely monitor how and when it is disseminated.

2.3. Scientific or scholarly background:

Practical challenges limit the success and generalizability of traditional behavioral interventions. Time commitments associated with traditional psychological interventions like cognitive behavioral therapy (CBT) can be prohibitive for adolescents. The average refusal rate for CBT in adolescent chronic disease settings is 37%; subsequent attrition is up to 32%, meaning over half of patients offered CBT fail to initiate or complete the intervention.[46] While many interventions designed for adolescents with T1D have been reported,[37,38,47,48] most fall within the traditional CBT model requiring in-person sessions spanning several weeks, and facilitation by a mental health professional with an advanced degree. These limit the generalizability of who is able to enroll and complete all sessions, and also impacts the overall cost-effectiveness. Shorter skills-based interventions with booster content delivered remotely may be more successful in adolescents.[49,50]

Stress and coping theory[42] provides an excellent model for intervention development. The stress and coping theory suggests three categories of resources to reduce distress and maintain well-being during illness: 1) dispositional factors, 2) situational factors such as stress management and goal-setting, and 3) coping process to create positive meaning (i.e., cognitive reframing).[44] Each of these impacts appraisals of stressors and the means to cope effectively with stress. While dispositional factors may be difficult to change, the Promoting Resilience in Stress Management (PRISM) intervention will teach the skills aligned with situational factors and coping processes which will help participants appraise disease-related stressors in a more adaptive way and use more effective coping strategies which we hypothesize will lead to improved disease outcomes. "Disease outcomes" in our case includes both A1C and diabetes distress and the

original figure should not have included the arrows leading from one to another.

Revised Figure 1. Schematic of theoretical framework guiding PRISM intervention's impact on proposed outcomes.

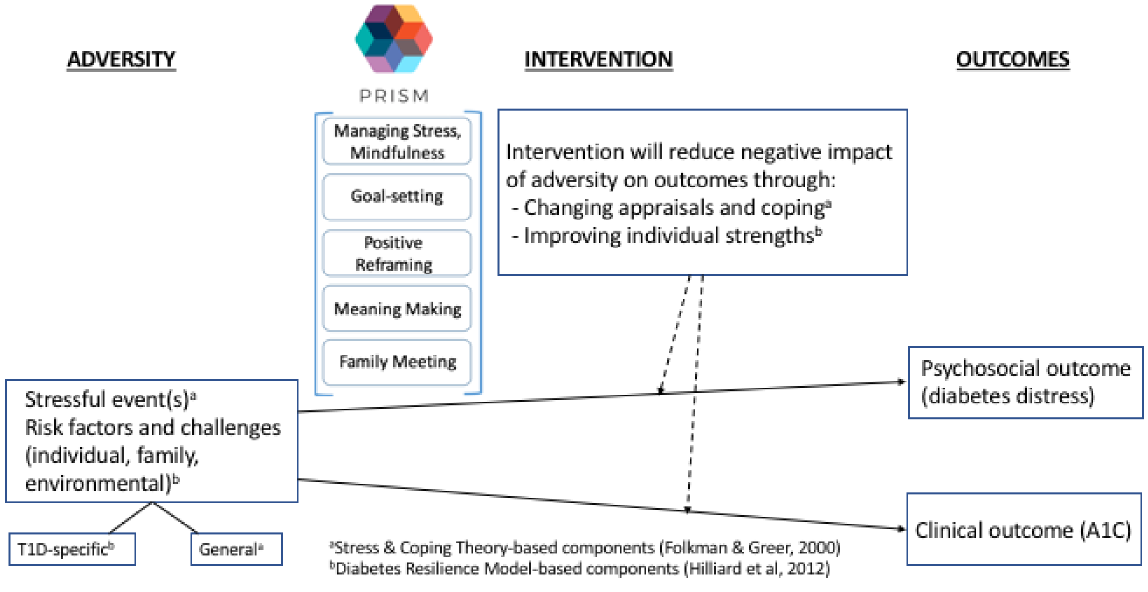

In addition to stress and coping theory, the proposed intervention aligns with the Diabetes Resilience Model.[20] Developed by co-I Hilliard, this diabetes-specific model of resilience for adolescents with T1D posits that enhancing individual strengths (referred to in this proposal as "resilience resources") helps adolescents manage stressors and risk factors (diabetes-specific and general sources of stress) in order to achieve positive outcomes. The positive outcomes include psychological/behavioral outcomes (e.g., diabetes distress) and clinical outcomes (e.g., A1C). This diabetes-specific model complements the stress and coping theory as outlined in the revised Figure 1. Our analytic strategy reflects this figure.

2.4. Prior approvals:

N/A

3. Study Endpoints[2]

3.1. Primary and secondary endpoints:

A1C was selected as the primary outcome as it is the gold standard of overall diabetes-related health status and for its known association with diabetes distress.[1,9,11] Given the known limitations of A1C as a clinically meaningful outcome, however, we will also evaluate participant time in range (70-180 mg/dl) for those using CGM as it is more specific and sensitive than traditional A1C testing (i.e. an intervention that addresses acute instances of hypo- or hyperglycemia may be detected in a time in range assessment but not in an A1C assessment).[51] Additionally, time in range may be more likely to associate with PROs compared with A1C given its overall representation of patient experience.[51] Diabetes distress was selected given strong evidence that distress is linked

with A1C and complications[1,9,11] and also because our pilot experience suggests PRISM was associated with reduction of distress.[31]

3.2. Primary or secondary safety endpoints:

n/a

4. Drugs, Devices and Biologics[3]

4.1. Manufacturer and name of all drugs, devices and biologics:

n/a

4.2. Description and purpose of all drugs, devices and biologics:

n/a

4.3. Regulatory status of all drugs, devices and biologics:[4]

n/a

4.3.1. Drugs or Biologics:

☐ IND Exempt. Explain:[5] ✍Click here to enter text.

☐ IND.

4.3.2. Devices:

☐ IDE Exempt. Explain:[6] ✍Click here to enter text.

☐ Abbreviated IDE / Non-Significant Risk. Explain:[7] ✍Click here to enter text.

☐ IDE / Significant Risk.

4.4. Plans to store, handle, and administer any study drugs, devices and biologics so they will be used only on subjects and be used only by authorized investigators:

n/a

5. Procedures Involved

5.1. Study design:[8]

This is a 4-year, multi-site RCT of PRISM compared to usual care among adolescents with T1D and clinically elevated distress scores (PAID-T≥30[7,52]). We intend to enroll 250 adolescents and after ensuring they meet the rest of the eligibility criteria, expect to randomize 160-176 people. We will use a stratified randomization scheme with 1:1 allocation to PRISM or usual care (n=80-88 each). Randomization will be stratified by sitePRISM-assigned adolescents will be scheduled for two PRISM standard sessions (one-on-one skills-based sessions directed at stress-management, goal-setting, positive reframing, and meaning-making), followed by one "Coming Together" session (facilitated family-meeting during which the adolescent may share what was learned). All sessions will be offered in-person, by web-based video chat, or over the phone at a time interval of every 2 weeks. Boosters (1:1 check-in sessions with the interventionist probing about progress and roadblocks) will be delivered every 2 weeks until 3-months post enrollment, at which point they will be offered monthly until the end of month 6. The digital app (and/or paper worksheets) will be available throughout the intervention, but will not include individualized communication through the app. Surveys will be collected from adolescents in both arms upon

enrollment and every 3 months for 12 months. Finally, A1C will be collected at each clinic visit and continuous glucose monitor (CGM) data (where applicable) will be collected.

5.2. Research procedures:[9]

Intervention Protocol and Procedures. Upon enrollment, participants will complete baseline questionnaires and an Hba1c dried blood spot at-home test kit (if A1c is not available in the chart in the prior 4 weeks) and then will be randomized 1:1 to PRISM or usual care. Patients will receive a study schedule that outlines study procedures based on whether they are assigned to PRISM or usual care. Standard psychosocial support (usual care) will be offered regardless of randomization. In order to provide the blood spot, the participant or their parent will need to do a finger poke using the lancet provided within the test kit. The blood collection lancet is an FDA 510K cleared device, which is what the patient will use to poke their finger and collect the blood on the paper. The test itself is cleared by the FDA as a moderately complex test (under lab test regulations). The blood spot cards we will be utilizing (Whatman 903 cards) are approved by the FDA as Class II devices.

PRISM Intervention. The total intervention consists of two, approx. 45-90 minute, one-on-one sessions approximately 2-4 weeks apart. The goal of the intervention is to teach resilience resource skills for use in current or future stressful situations. Each session covers two topics as described in **Table 1**. A 3rd session follows, which includes both the adolescent and participating caregiver(s) ("Coming Together"). Although the intervention framework is not disease-specific, diabetes-specific examples will be provided for each topic to enhance the relevance of the skills to participants' daily diabetes-related stressors. A trained research associate (RA) conducts the intervention, as described in previous models and our pilot studies.[29,53] The 1st session occurs within 2 weeks of enrollment, ideally in-person (alternative formats listed below) and in conjunction with the enrollment visit. The 2nd and 3rd sessions are scheduled approximately every 2-4 weeks around the participant's preferences. There can be flexibility with combining or separating PRISM skills based on patient preference (e.g., participant becomes fatigued after stress management component, goal-setting can occur during a separate scheduled time). When visits cannot be conducted in-person, alternatives include web-based video chat (i.e., webex, skype, zoom, whatsapp), or by phone/conference call. Following the 3rd session through week 12, participants receive bi-weekly "booster" contacts (1:1 check-in sessions by telephone with the interventionist) to practice/refresh skills and check-ins on how skills have been utilized. These boosters will then be delivered monthly in months 4-6. In addition, all AYA PRISM participants have access to the digital PRISM app, which offers an interactive practice and tracking interface to continue enhancing skills. While the app provides additional opportunities to practice the skills they have learned in-person, there is no personalized communication provided in the app between the interventionist and participant. The participant is not required to use the app (paper-pencil worksheets are also available for those who do not want to use an app).

Interventionist Training and Fidelity. PRISM has been standardized through the creation of comprehensive interventionist protocols. All interventionists undergo at least 4 hours of in-person training including role-playing, dynamic feedback, and mock sessions. The fidelity of sessions will be systematically assessed via audio-recording. Any session can be audio-recorded with the participant's permission. A PI or lead interventionist will review the first 3, and then 1 in 6 randomly selected sessions conducted by each interventionist throughout the course of the interventions. Recordings will be scored for fidelity with a standardized tool. Interventionists will receive

feedback regarding adherence to protocol and approach will be refined as needed. A lead interventionist will provide debriefing counsel to all interventionists. The lead interventionist will also supervise cross-site calls on a monthly basis to problem-solve and discuss fidelity challenges to help standardize interventionist responses to the unexpected.

Usual Care. Families in both randomization arms will receive usual medical care for diabetes, including access to psychosocial care provided by the mental health professionals affiliated with the diabetes clinic if needed. At SCH, screening for diabetes distress and depression using standardized tools is performed for all patients 13 years old and above and social work is offered on an annual basis. TCH standard care includes routine depression symptom screening by diabetes care providers, as well as at least annual social work assessments including interview-based screening for psychosocial stressors and diabetes-related distress. For both sites, every diabetes patient is cared for by a team of diabetes specialists which includes a provider (MD, Physician Assistant and/or Nurse Practitioner), diabetes educator, and social worker. Subspecialty referrals for additional mental health, nutritional or other support are made at the discretion of the primary diabetes provider.

Upon completion of the study, all participants in the usual care arm will be given access to the PRISM app. The app contains self-directed teachings of the 4 pillars but there is no personal teaching or check-ins from an interventionist. The app can be used indefinitely.

5.3. Data sources that will be used to collect data about subjects:[10]

Instruments. The objective of this proposal is to determine the clinical value of the PRISM intervention among adolescents with T1D and their families. Specifics for the proposed measures, by Aim, are below and in **Table 2a and 2b**.

Aim 1 Measures. HbA1C is collected at every in-person clinic visit as standard of care across both sites from trained medical staff drawing blood samples using the fingerstick method. Blood assays are analyzed immediately using the DCA 2000 Hemoglobin HbA1C system (Siemens-Bayer). Patients with virtual clinic visits and/or no Hba1c recorded within the prior 4 weeks will collect a dried blood spot sample and mail to the central in advance of their appointment. The HbA1c on filter paper is extracted and measured on the Vitros 4600 HbA1c assay. This assay is correlated with the DCA 2000. Although we will not have a central lab, both sites are using the same point-of-care machines and lab tests which alleviates the cost-burden of a central lab and also mimics other large, multisite studies (e.g., T1D Exchange and SEARCH for Diabetes in Youth).HbA1C will be tracked at each visit after enrollment from their medical record or an at-home A1c blood spot test kit will be provided for the patient, which has been routinely incorporated into care at Seattle Children's since May of 2020. Given recent consensus reports recommending attention to additional glycemic metrics beyond HbA1c, for those patients using CGMs, we will also collect time in range (percentage of readings in the range of 70–180 mg/dL).[51] All CGM data will be downloaded using standard CGM device-reading software available.Patients will be asked to share their CGM code with us the same way they do with their providers, which is part of usual clinical care.

**Table 2a. Data collection instruments (Adolescent)**			
**Aim**	**Construct**	**Measure**	**Source (and Time in months)**
1	Glycemic control	HbA1C	Chart review or At-Home A1c Kit
	Time in Range	CGM download	Device download
	Patient Demographics	PRISM Diabetes Patient Demographics	Assessed only at BL
	Med Info	Updated Med Info Form	
2	Diabetes distress	PAID*	(at BL, this is the screening questionnaire)
	Resilience	CD-RISC*, DSTAR*	
	QOL	T1DAL	Adolescents (BL, 6 and 12 months)
	Adherence	SCI-SF<br>DSMQ	
	Family Conflict	DFCS-R	
		PHQ-8	
	Depressive Symptoms	BFSC	
	Benefit-Finding	The COVID-19 Impact	
	COVID-19 Impact	Questionnaire	
		READDY	
	Transition Readiness		Assessed only at BL and 12 months.

CGM=Continuous Glucose Monitor, BL=Baseline(see below for survey names).
**Also assessed at 3 and 9 months*

**Table 2b. Data collection instruments (Parent)**			
**Aim**	**Construct**	**Measure**	**Source (and Time in months)**
	Parent Intake Form	PRISM Diabetes Parent Demographics (BL only)	
2	Diabetes distress	PAID	Parents (BL, 6, and 12 months)
	Resilience	CD-RISC	
	QOL	T1DAL	
	Adherence	SCI-SF DSMQ	
	Family Conflict	DFCS-R	
	Family Impact	DFIS	
	COVID-19 Impact	The COVID-19 Impact Questionnaire	

BL=Baseline(see below for survey names)

Aim 2 Measures. Diabetes distress will be assessed with the Problem Areas in Diabetes Scale (Teen Version) (PAID-T), a 14-item scale assessing the perceived emotional burden of living with diabetes.[7,52] Clinically meaningful cut-points have been reported[52] and this tool is used as part of clinical care in the SCH Diabetes Clinic. It is the only measure of diabetes distress developed and validated purposely for use with adolescents and measures emotional distress related to the challenges of living with type 1 diabetes more specifically than measures of general distress or depressive symptoms. The parent-version will be administered to assess parental distress about his/her child's diabetes. Self-reported resilience will be measured with the 10-item version of the Connor-Davidson Resilience Scale (CD-RISC).[54,55] This instrument has excellent psychometric properties and has been validated and used in adolescent populations,[56] including in diabetes.[29] Adolescent and parent participants will each complete this scale assessing their own resilience. Adolescents' diabetes-specific "strengths" (i.e., positive behaviors and attitudes related to the challenges of living with T1D) will be assessed with the Diabetes Strengths and Resilience measure (DSTAR), a 12-item self-report measure with strong psychometric properties.[41] Only adolescents will complete the DSTAR, and there are two versions (one for 13-17 and one for 18+). Adolescent benefit-finding will be measured with the Benefit Finding Scale For Children (BFSC),[57] which is a 10-item instrument with good psychometric properties, which has been utilized to assess benefit-finding in pediatric patients, including adolescents with type 1 diabetes.[58] Adolescents will complete the BFSC. Adolescent depressive symptoms will be measured with the 8-item Patient Health Questionnaire (PHQ-8), a widely used instrument among general populations and patients with chronic illness.[59-61]This instrument has excellent psychometric

properties.[60,62] The adolescents will fill out the PHQ-8. Diabetes-specific quality of life will be assessed using the adolescent self-report version of the Type 1 Diabetes and Life (T1DAL) Measures which assesses diabetes-specific health-related quality of life (HRQOL) for people with type 1 diabetes (T1D). Parents will complete the parent-version which assesses their own HRQOL.[63] The Self-Care Inventory-Short Form (SCI-SF) and the Diabetes Self Management Questionnaire (DSMQ) will be used to assess self-reported adherence. The SCI-R is a 14-item measure assessing perceptions of adherence to diabetes self-care recommendations over the previous 1–2 weeks, which has been adapted to a 9-item shorter form to include only the aspects of adherence that relate to diabetes control[64,65] SCI-R assesses four domains of adherence behaviors (monitoring, insulin, diet, and exercise) and has been consistently reported as a meaningful measure of self-reported adherence.[65-67] In collaboration with the authors of the SCI-R, this measure has been abbreviated and slightly modified to reflect current diabetes technology. Adolescents will complete a self-report version of the modified SCI-R (i.e., the SCI-SF), and parents will complete a parent-report version to assess parent's perspectives of their child's adherence. The DSMQ is a validated 9-item questionnaire which assesses adherence to diabetes self-management tasks.[68] Adolescents will complete a self-report version of the DSMQ, and parents will complete a parent-report version. The DSMQ has been used as a brief measure of self-management in studies of pediatric patients with type 1 diabetes.[69] Family conflict and family impact will be used to assess the burden of diabetes on family dynamics using validated tools. Family conflict (Diabetes Family Conflict Scale, Revised, DFCS-R) is assessed from both the parent and adolescent perspective, and family impact (Diabetes Family Impact Scale, DFIS) is assessed only from the parent perspective.[70,71] Transition-readiness is relevant to this age group and will be assessed with the READDY, a tool that is used in the diabetes clinics to assess readiness to transition to adult care.[72] Adolescents only (not parents) will complete the READDY. The COVID-19 Impact Questionnaire is a 21-item, self-report questionnaire developed for this study to assess perceived impact of the COVID-19 on adolescents and parents. Items assess worry/anxiety related to COVID-19, life events as a result of COVID-19 (e.g., loss of job, missed school), lifestyle changes (e.g., social distancing), known COVID-19 symptoms/diagnoses/treatment of self and family members, and perceived impact of COVID-19 on diabetes management.

Additional covariates. Demographic and clinical characteristics as well as information about healthcare utilization and medical technology updates will be assessed by chart review and/or self-report including the participant's diagnosis date, insulin regimen, use of CGM, comorbidities, insurance coverage, medications, and other patient demographics. We will assess family size, parent employment, education, race/ethnicity, insurance coverage, and other socio-economic indices through the baseline questionnaire. Patient and parent demographics will be collected at baseline only, and medical technology changes relevant to diabetes management will be assessed every 3 months.

Time from previous PRISM session	Within 4 weeks of enrollment	2-4 weeks after PRISM 1	2-4 weeks after PRISM 2	Every 2 weeks after PRISM 2 through the end of month 3	Once a month (Months 3-6)	
**Session Schedule** (PRISM Group only)	PRISM 1	PRISM 2	PRISM 3	Boosters	Boosters	

Time from enrollment	0	3 Months	6 Months	9 Months	12 Months
**Survey Schedule** (both arms)	Survey	Survey	Survey	Survey	Survey
**A1C** (both arms)	At-Home Test Kit (if not in Chart)	From Chart (If Available)	At-Home Test Kit (if not in MR)	From Chart (If available)	At-Home Test Kit (if not in Chart)
-------Medical Record Abstraction-------<br>-------Continuous Glucose Monitoring (GCM) and A1C as Available-------					

5.4. Data to be collected, including long–term follow–up data:[11]

Data collection and management. Systematic data collection, quality control, and data-management procedures will be implemented including: (1) protocols for data collection; (2) rigorous training and oversight of study staff with ongoing monitoring of adherence to protocols; (3) regular review of questionnaire response rates and missing items to identify and correct problems; (4) verification of all data through computerized data entry systems restricting invalid/out-of-range responses; and, (5) weekly team meetings and progress reports to provide feedback to study staff concerning difficulties and follow-up to ensure problems are resolved quickly.

A1C and CGM data: Study staff will take extra steps to ensure primary outcome data is obtained. This will include careful tracking of all participants to ensure regular clinic visits are scheduled and attended, and reminder phone calls and texts prior to the appointment. In cases where the 3-month window is passed, participants will be reminded to schedule their Standard of Care appointment and/or transferred to Seattle Children's scheduling department. If patients are not scheduled for a standard care appointment and/or the clinic is not offering labs due to COVID-19, an at-home A1c test kit will be mailed to the families to ensure collection of A1c data. Families will return mailed kits to the CLIA-Certified laboratory at each respective institution (i.e., Seattle Children's or Texas Children's).

Survey data: To optimize collection, all participant surveys will be available by paper or online via the REDCap system, a secure HIPAA-compliant, high-quality data collection tool. To ensure data quality, a RA will review surveys for their completion for missing fields or this will be set up for automation within RedCap.

Medical record data: RAs will collect and upload CRF data quarterly during the 12 months the patient is on study and will include a CRF for the previous diabetes-related visit in closest proximity to the date of enrollment (in instances in which the patient is not seen by a provider during the enrollment period). To ensure reliability and validity of abstracted medical record data, we will use our current methods for training and quality control, including guided practice abstraction and independent abstraction with reconciliation by a medical Co-I (DeSalvo/Malik). A 10% random sample will be dual abstracted.

6. Data and Biospecimen Banking[12]

6.1. Complete list of the data and/or biospecimens to be included in the bank:[13]

Surveys, CRFs (e.g., medical record data), a1c results, and audio transcriptions will be coded and labelled with study ID numbers by the study team.

6.2. Location of data and/or biospecimen storage:[14]

All will be stored in original (hard copy) forms in locked cabinets (where relevant) and/or password-protected secure databases (for all electronic survey data). At-home test kit A1c data will be stored per laboratory protocols at each respective institution.

6.3. List of those with direct access to data and/or biospecimens in the bank:

Data will not be shared outside the group of investigators conducting the study at SCH and BCM/TCH. When other investigators are interested in new analyses, the PIs will verify they have IRB approval to conduct additional analyses and will be added to the study team. Coded study data will be banked indefinitely for future use by the group of investigators conducting the study and access will be controlled by the PI's. Future studies will formally test the dissemination and implementation potential of the intervention once its efficacy is confirmed.

6.4. Length of time data and/or biospecimens will be stored in the bank:

Original surveys will be saved for 10 years or until final analyses are completed, whichever occurs last, in order to ensure data quality. Coded electronically saved study data will be banked indefinitely for future use by the group of investigators conducting the study and access will be controlled by the PIs.

6.5. Procedures for protecting the confidentiality and privacy of the subjects from whom the data and/or biospecimens were collected:[15]

Per 6.1, all banked data will be coded and labelled with only study ID numbers, not identifying information.

6.6. How the data and/or biospecimens will be made available for future use:

6.6.1. Who can request data and/or biospecimens from the bank:

Investigators interested in conducting analyses will need to contact the PI and have IRB approval to conduct analyses.

6.6.2. Format in which data and/or biospecimens will be provided:
Deidentified/coded study database.

6.6.3. Process for investigators to request data and/or biospecimens:[16]
Contacting study PI.

6.6.4. Restrictions on future use:[17]
There are no restrictions on future use of de-identified, banked data.

6.6.5. Plan for providing data results from banked data/biospecimens:
Results of initial analyses will be shared with participants if participants request on the consent form that they would like a summary of study results. A1C data will be placed in patient's chart at Seattle Children's and sent to individual providers at BCM/TCH with oversight from study site endocrinologists Malik and DeSalvo for each respective institution.There is no plan for results from banked data to be shared with participants.

7. Sharing of Results

7.1. Plan to share results with subjects/others:[18]

For Phase 1 (Screening), data will not be shared outside the group of investigators conducting the study at SCH and BCM/TCH. If other investigators are interested in analyses with screening data, the PIs will verify they have IRB approval to conduct analyses. Coded screening data will be banked indefinitely for future use by the group of investigators conducting the study and access will be controlled by the PIs. Participants will not be informed of their specific PAID-T results in Phase 1, however, participants will be immediately made aware if they are eligible for Phase 2 based on their survey results.

For Phase 2 (Full Study), at SCH, data collected from at-home A1c test kits will be entered into the patient's chart as a lab that was performed and will be available to clinical providers (as this same test is standard of care for tele-health visits at SCH). At BCM/TCH, data collected from at-home A1c test kits will be sent to individual providers via EPIC with a description of the A1C test, how it was collected, and BCM/TCH laboratory validation (since this test is not standard of care at BCM/TCHPatients and parents will be notified of this during the consent conference. Other data will not be shared outside the group of investigators conducting the study at SCH and BCM/TCH.When other investigators are interested in new analyses, the PIs will verify they have IRB approval to conduct additional analyses. Coded study data will be banked indefinitely for future use by the group of investigators conducting the study and access will be controlled by the PI's.   Future studies will formally test the dissemination and implementation potential of the intervention once its efficacy is confirmed. If indicated by participants, a summary of the primary analyses will be shared at the study closure.

8. Study Timelines

8.1. Duration of an individual subject's participation in the study:
12 months

8.2. Duration anticipated to enroll all study subjects:
18 months

8.3. Estimated date for the investigators to complete this study:
March, 2023 (4 years)

**Timeline of research activities**																
	Year 1				Year 2				Year 3				Year 4			
**Study Implementation Activities**	Q1	Q2	Q3	Q4	Q1	Q2	Q3	Q4	Q1	Q2	Q3	Q4	Q1	Q2	Q3	Q4
	Apr '19	Jul '19	Oct '19	Jan '20	Apr '20	Jul '20	Oct '20	Jan '21	Apr '21	Jul '21	Oct '21	Jan '22	Apr '22	Jul '22	Oct '22	Jan '23
Study set-up and PRISM training at both sites	■	■	■													
**Aims 1 and 2: PRISM Intervention and Data Collection of A1C and PRO questionnaires**																
Recruitment, Enrollment, Randomization, and Delivery of PRISM				■	■	■	■	■	■							
Follow-up (6-months of PRISM boosters and 12-months of questionnaire and A1C tracking)					■	■	■	■	■	■	■	■	■			
Aims 1 and 2 data analysis												■	■	■	■	■

9. Study Population[19]

9.1. Inclusion criteria for each subject population (e.g., patients, parents, providers):
We will recruit adolescents and their caregivers from diabetes clinics at SCH and BCM/TCH.

Adolescents will be eligible if:

- They are 13-18 years old
- Diagnosed with T1D >=12 months
- Elevated distress score (PAID-T>=30)***** (within the prior 12 months)
- Speak English fluently
- Cognitively able to participate in intervention sessions and complete written surveys.

*****Please note that at BCM/TCH, screening for distress is not Standard of Care. Therefore, we will complete a screening informed consent (if patient is present in clinic) or an information sheet (if patient is not present in clinic) for patients at BCM/TCH that meet all other criteria and then

proceed with the PAID-T screener (via paper/pencil, verbally, or REDCap). We call this "Phase 1" of the consenting process. In Phase 1, PAID-T will only be completed by adolescents.

If interested, those with an elevated distress score (PAID-T>=30) will move onto the full informed consent process, will be enrolled on the full study (i.e., "Phase 2" of the consenting process), and will go on to complete baseline questionnaires and be randomized. Those who complete the screening informed consent, take the screener (i.e., the PAID-T) and are not eligible for "Phase 2" of the consent processes (i.e., PAID-T < 30) will be given a $5 gift card. They will not complete the baseline questionnaires or be randomized. At SCH, this process will be followed if a current PAID-T (i.e., administered within the prior 12 months) is not available at the time of consent. For all who complete the PAID-T screener as part of "Phase 1" consenting process, PAID-T score will be used as the baseline value.

Caregivers will be eligible for the survey completion if:

- Adolescent of parent or guardian agrees to participate in study
- Adolescent participant provides verbal assent or verbal consent if 18 or over for parent or guardian to complete surveys.
- Parent/Guardian is cognitively and physically able to participate
- Parent/Guardian is able to speak and read English or Spanish language
- Parent/Guardian participant has signed informed consent for study participation

Caregivers will be eligible for the "Coming together" session if:

- Adolescent of parent or guardian agrees to participate in study
- Adolescent of parent or guardian has been randomized to the PRISM intervention arm of the study
- Adolescent participant provides verbal assent or verbal consent if 18 or over for parent or guardian to complete 'Coming together' session
- Parent/Guardian is cognitively and physically able to participate
- Parent/Guardian is able to speak English or Spanish language
- Parent/Guardian participant has signed informed consent for study participation

9.2. Exclusion criteria for each subject population:

Adolescents are excluded if:

- Patient refusal to participate (any age), or parental refusal to participate for patients less than 18 years of age
- Cognitively or physically unable to participate
- Patient unable to speak in the English language
- Patient unable to read in the English language
- Patient is a ward of the state
- Severe comorbidities including other major chronic health conditions that significantly impact daily management demands or health outcomes.
- Prior participants of PRISM studies

Caregivers are excluded if:

- Adolescent refusal to participate
- Parent/Guardian/Caregiver is <18 years of age
- Parent/Guardian/Caregiver is unable to read English or Spanish.
- Parent/Guardian/Caregiver is unable to speak in English or Spanish
- Parent/Guardian/Caregiver is cognitively or physically able to participate.

9.3. Populations with special considerations, involved in the study:[20]

☒ Children/Teenagers[21]
Risk assessment specific to this vulnerable population and additional safeguards:[22]

Individuals who are not yet adults (children, teenagers): Pediatric patients with serious illness are at risk for poor outcomes and may benefit from resilience-enhancing interventions in the future. We justify the inclusion of children in this project because the implementation of those interventions requires feasibility information and patient feedback. This study will provide those crucial data. Patients enrolling in this study may, in fact, benefit from the intervention; however, at the time of consent, we will ensure that all patients and families understand the objective of this study are to test the feasibility of this intervention such that it may be used prospectively in the future (see risks/benefits above).

☐ Children who are Wards of the State[23]
Risk assessment specific to this vulnerable population and additional safeguards:
✍Click here to enter text.

☐ Adults Unable to Consent [24]
Risk assessment specific to this vulnerable population and additional safeguards:
✍Click here to enter text.

☐ Neonates of Uncertain Viability or Non–Viable Neonates[25]
Risk assessment specific to this vulnerable population and additional safeguards:

✍Click here to enter text.

☒ Pregnant Women[26]
Additional safeguards:

Pregnant women: There is a chance that a parent/caregiver of a patient participant is pregnant and therefore could participate in the survey and/or 'coming together' portion of this study. This study does not involve interventions/invasive procedures to the woman or fetus and does not involve fetuses or neonates as subjects.

☐ Prisoners[27]
Additional safeguards:
✍Click here to enter text.

☐ Economically or educationally disadvantaged persons[28]
Additional safeguards:
✍Click here to enter text.

10. Number of Subjects

10.1. Total number of subjects to be enrolled locally:[29]
N=90

10.2. Total number of subjects to be enrolled across all participating sites:[30]
N=250

10.3. Number of screened subjects versus the actual number enrolled in the research:[31]
Using the eligibility criteria age, A1C, and language against our existing databases at SCH, we estimate over 200 eligible adolescents will be screened at each of the two sites.

10.4. Power analysis:

Table 3. Detectable differences in select outcomes between groups given 80% Power and 5% Type I Error Rate

Aim	Outcome	Projected mean for control group	Standard Deviation	Detectable difference in mean
1	A1C	9.1	1.9	1.1
2	PAID-T	49.3	16.2	9.2

Our focus for sample size estimation is the primary outcome (Aim 1): A1C at 6-months. Sample size is based on data from the T1D population relevant to age and distress levels, suggesting A1C scores are normally distributed with a mean of 9.1±1.9.[7,52] Assuming 20% attrition based on prior trials,[29,31] we will randomize 160-176 adolescents (80-88 per arm) to obtain an evaluable total sample size of 100 participants (50 per arm). This sample size achieves 80% power to detect a minimum difference of 1.1 in the mean 6-month A1C, based on two independent samples t-test. Minimally detectable differences for A1C and diabetes distress (PAID-T) based on 50 subjects per arm are shown in **Table 3**.

11. Withdrawal of Subjects

11.1. Anticipated circumstances under which subjects will be withdrawn from the research without their consent:

In this intention-to-treat analysis plan, all participants who fill out a baseline survey and have a baseline A1c (either collected through medical record or A1c at-home test kit) will be included in analyses. As above, withdrawal will be determined by study staff only in the event of medical complications or death. We may also withdraw patients who do not complete surveys or an A1c within six weeks of enrollment. In rare instances in which the patient/family encounters a mailing issue, we will extend a 2-week grace period. Subjects who withdraw will be asked to complete a short survey which will simply be a question as to why they have decided to withdraw from the study.

11.2. Procedures for orderly termination:

We will inform the family with a phone call from an RA or investigator (followed up by a written letter or email from the PI) that they are no longer on study.

11.3. Procedures that will be followed when subjects withdraw from the research, including partial withdrawal from procedures with continued data collection and withdrawal from data/biospecimen banking:

Response to the intervention will be determined upon study completion (not in “real-time,”). All participants, including those on the intervention arm, will continue to receive usual care, including as-needed referrals for professional psychology services.

All participants may choose to withdraw from the study at any time. We will track date of discontinuation and request a brief reason to be recorded for tracking purposes. To ensure primary outcome data, we will offer an abbreviated survey with only the PAID and CD-RISC for those who do not complete all assessments or who request to drop out.

In the event of serious medical complications (or death) precluding participation, participants will be censored after 2 months of missing data. In all other cases (non-completion of surveys without serious medical complications or death), staff will continue to request surveys or at-home A1c test kits until 6 weeks following each timepoint.

Unless explicitly indicated by participants who withdraw their consent, data collected from participants up to the time of withdrawal will be maintained and utilized in analyses, and study staff will continue to request follow-up surveys as determined by the protocol. For participants who request abbreviated survey packets, we will oblige (see section 14.2).

12. Risks to Subjects

12.1. Reasonably foreseeable risks to subjects (include each study population, each arm, and optional procedures):

- **Adolescents with high distress and/or depressive symptoms.** Patients who screen for high distress and/or high depressive symptoms regardless of their decision to enroll in the study may have their scores shared with a member of their local diabetes team per departmental standard operating procedures. The diabetes provider will use relevant clinic-specific procedures and his/her discretion to determine what next steps, if any, are indicated (e.g., social work consult, referral to psychology) with the family. We may also reach out to the patient and/or parent to check in and/or provide community/hospital resources.
- **Adolescents with high A1c.** The study endocrinologist (Malik (SCH) & DeSalvo (BCM/TCH)) will be alerted if a patient takes an at-home A1c test for research purposes and score >14%. The study endocrinologist will then contact the patient's primary diabetes provider to alert the provider of this patient's score, and the diabetes provider will use relevant clinic-specific procedures and his/her discretion to determine next steps.
- **A1c At-Home Test Kit.** Patients would be asked to do a self needle poke on three occasions (Baseline, 6 months, 12 months) in order to complete their A1c at home test kits. When doing a needle poke, some people may get a small bruise that will go away in a day or two. Some people may feel dizzy or faint. There is also a very small chance that the patient could get an infection where the needle pokes the skin.These risks will be outlined in the consent documents and potential participants would be made aware of these risks during the consent conference.
- **PRISM.** The intervention ("Promoting Resilience in Stress Management", PRISM) and our surveys may address sensitive matters in that it they patients to identify stressors and negative thoughts. Adolescent participants may be prompted to think about the threat to their future posed by their diabetes. During the coming together session and/or while completing surveys, parent(s)/caregiver(s) may be prompted to think about the threat to their child's future by diabetes. The topics to be covered may provoke sadness, anxiety, depression, fear or doubt for adolescents and/or their parent(s)/caregiver(s). Administrators of the intervention will be trained to immediately inform the patient's primary social work and/or medical teams if the patient and/or parents/guardians endorses thoughts of self-harm or harm to others, per the clinic policy for responding to suicidal ideation. As part of their informed consent process, participants will be made aware of this policy, as well as the fact that confidentiality may be broken in the case that providers see an immediate threat to the patient's or another's safety. No physical risks are expected to arise from the study.

  The primary risk to participants will be concerns about confidentiality, and stress of discussing the topic of their or their child's diabetes experience. While the potential risks to participants are low, we will take steps to ensure that all potential risks are handled appropriately as described below.

  Due to the nature of the PRISM intervention, all staff and participants will be unblinded from the time of randomization.

We recognize the unique risks of data collection for an adolescent population. The major risk is compromise of personal data. Thus, confidentiality procedures for all data will be a priority for this study. All data will be maintained on secure servers accessed by secure computers or in locked offices at Seattle Children's Research Institute (data collection center) or in CLIA-Certified laboratories at SCH and BCM/TCH (for A1c data). Data and paper materials will be stored locally at BCM/TCH as well. The study results will be kept for at least ten years or until final analyses are completed, whichever occurs last, in order to ensure data quality. The subject's consent to use or share PHI does not expire. Access to the buildings where the study data will be kept at Seattle Children's Research Institute and BCM/TCH are limited to authorized personnel. The Lead RA and other researchers involved in this project have years of experience and has received ongoing training at Seattle Children's Research Institute or BCM/TCH on confidentiality as well as HIPAA confidentiality standards. Our and other previous trials have kept their data at Seattle Children's Research Institute and BCM/TCH in the recent past, and the security and confidentiality of the data have never been compromised.

12.2. Procedures with unforeseeable risks:

Although this study is considered low risk, the study team has extensive experience running the PRISM intervention, and we have not experienced unforeseeable risks. IF unanticipated risks come to the attention of study staff, the DSMC will be alerted and if concurrent with federal law, IRB rules, and/or the investigator manual/SOPs, will be reported as needed to the appropriate group(s) (i.e., IRB, DSMC, PI, etc.).

12.3. Procedures with risks to an embryo or fetus should the subject be or become pregnant:

n/a

12.4. Risks to others who are not subjects:

n/a

12.5. Procedures performed to lessen the probability or magnitude of risks:

To mitigate these risks, we will emphasize the voluntary nature of the study to adolescents and parents and that the study will in no way impact or influence clinical care. We will let adolescents know that their information will be kept confidential and that transcripts will be de-identified and audio recordings destroyed after transcribing. We will let patients know that their A1c data may be available to their providers. We will let adolescents know that they can change their mind about participating and may decide to withdraw from the study at any time.

Each of the participating sites has significant experience with clinic recruitment for studies and has developed processes to ensure best practices with each study participant recruitment. These procedures and scripts also emphasize that any potential coercion on the part of parents should not take place, and that adolescents' decision for or against participation in the study does not affect the clinical care they receive.

The investigators and staff will be available to answer any questions from potential participants via phone or email throughout the study. We will emphasize that the decision of whether or not to be part of this study does not affect their ongoing care at their respective institutions.

13. Potential Benefits to Subjects

13.1. Potential benefits that individual subjects may experience from taking part in the research:[32]

We hypothesize that patients who receive the intervention will have better clinical and psychosocial/behavioral outcomes. However, there may be no direct benefit for participating in this study if our hypotheses are wrong. More broadly, information gained from this study may heighten the understanding of the adolescent diabetes experience and elucidate strategies that foster resilience and promote better outcomes in this group of high risk patients. These strategies could be extended to the care of other adolescent patients facing other chronic diseases. This research has the potential to contribute to the research base concerning the promotion of optimal quality of life and mental wellness for all adolescent patients.

14. Data Analysis/Management

14.1. Data analysis plan, including statistical procedures:

Randomization. The randomization algorithm will be constructed by the study statisticians (Zhou and Bradford) using a permuted blocks scheme with varying block sizes; randomization will be stratified by site. Study staff will be blinded to allocation until after enrollment.

Analysis plan overview. The primary statistical analyses will be intention-to-treat to avoid confounding by non-random participant attrition. Clinical characteristics including A1C and time in range and items within the surveys will all be summarized at time of enrollment and at 3, 6, 9 and 12-month time points using descriptive statistics: frequencies and proportions for categorical variables, means and standard deviations for continuous variables, or median and inter-quartile range if distribution is skewed.Demographic characteristics will be collected and summarized at baseline. All analyses will be adjusted for site and baseline A1C, as randomization is stratified by these variables. In addition, we will assess for confounding by *a priori* identified variables associated with distress and A1C including age, gender, race, primary language spoken at home, parental education, insurance status. If any of these analyses suggest confounding or are clearly imbalanced between groups, subsequent analyses will be adjusted accordingly.

Aim 1 primary outcome is A1C at 6 months. Because the amount of change depends strongly on the initial value at baseline we will control for baseline A1C as a covariate in the regression. We will treat A1C as a continuous outcome and apply linear regression model to estimate the mean between-arm difference and 95% confidence interval. The regression models will be specified as follows:
$A1C \sim PRISM + baseline\ A1C + site + CGM + additional\ covariates,$ where A1C is the outcome at 6 months, PRISM is the intervention indicator, baseline A1C, site, CGM status and other unbalanced confounders are the adjusted covariates. The coefficient of PRISM captures the adjusted mean differences in A1C between intervention and control arms, and will be tested using Wald t-test. In addition to the cross-sectional analysis of A1C at 6 months, we will also conduct repeated measures analysis using linear mixed effects models to examine the trajectories of A1C. This allows us a closer inspection of how participants respond to PRISM over time and examine

whether the intervention benefits persist over time. The linear mixed regression models will be specified as follows: $A1C_{ij} \sim b_{0i} + PRISM_i + Time_j + PRISM_i \times Time_j + covariates,$ where $A1C_{ij}$ is the A1C level for subject *i* at time *j*, b_{0i} is the subject specific random intercept to account for within-subject correlation due to repeated measures, and $PRISM_i \times Time_j$ is the group-by-time interaction term. When $Time_j$ is modelled as a discrete variable and baseline is the reference time point, the coefficients of the group-by-time interaction estimate the amount of differences in changes at later time points relative to baseline. Significance of these coefficients will be assessed using confidence intervals, and p-values from F-tests based on ANOVA and Kenward-Roger method.[73]

For time-in-range (CGM data), we will use a 2 group comparison of the change from baseline in the percentage of sensor values in the target range (70-180 mg/dL) using a linear model adjusted for the baseline value and factors used to stratify randomization. All subjects with any CGM data will be included. Residual values will be examined for an approximate normal distribution. If values are highly skewed then transformation or non-parametric methods will be used instead.

Aim 2. Secondary outcomes include mean scores at 6-months for the PAID-T, CD-RISC, D-STAR, T1DAL , PHQ-8, BFSC, and SCI-R. We will model each outcome separately following a similar approach to the primary analysis for Aim 1.

Additional analyses and considerations. We will conduct a thorough process evaluation that includes: (a) intervention fidelity; and, (b) satisfaction queries. Multiple comparisons are a concern since we are collecting multiple measures from participants and are interested in several hypotheses. We minimize this problem by specifying a limited number of main hypotheses for each aim. The Benjamini-Hochberg procedure will be used to control the False Discovery Rate criterion at α=0.05 to correct for multiple testing in analyses that are not pre-specified.[74] Likewise, in manuscripts and presentations, we will report the number of tests performed and interpret results within this context.

Heterogeneity of treatment effects (HTE) analysis. We will conduct exploratory analyses for additional heterogeneity of treatment effects. This refers to the non-random, explainable variability in the direction and magnitude of treatment effects for individuals within a population as we may be interested in identifying subgroups that the intervention works particularly well or poorly. We plan to conduct HTE analyses mostly through subgroup analysis. Examples of factors, alone or in combination, that potentially could modify the intervention effects include baseline A1C, CGM, language at home, or engagement with the intervention. Regression models similar to those outlined for Aims 1 and 2 will be applied to subgroups defined by these factors. Note these analyses are confirmatory and should be interpreted with caution.

Considerations regarding missing data. Data may be missing due to patients/families skipping individual survey items, omissions in medical records, lack of follow-up, medical complications, or death. We will quantify the amount of missing data, evaluate the association of participant characteristics with missing data, and minimize bias and increase efficiency in the associations of interest by applying appropriate methods to account for missing data.[75-77] For example, for outcomes where missing at random (MAR) is a plausible assumption, we will use multiple imputation or inverse probability weighting, depending on the statistical model being considered. For missing not at random (MNAR) data, we will use sensitivity analyses. In all cases, we will assess the robustness of estimates due to assumptions.

14.2. Quality control procedures for collected data:[33]

For survey data, all participant surveys will be available by paper or online via the REDCap system, a secure HIPAA-compliant, high-quality data collection tool. The rationale for offering both paper-pencil and electronic versions is based on our prior experience where participants preferred the former,[78] which in turn facilitated more complete data collection. To ensure data quality, a RA will review for missing fields and call participants to clarify and query/complete individual missing items verbally. Data will be quality controlled by a double-checking mechanism (i.e., data double-entry via REDCap or double-entry/checking of manually entered data).

For medical record data, RA's will collect and upload CRF data at a frequency varying between 2 weeks and 6 months.To ensure reliability and validity of abstracted medical record data, we will use our current methods for training and quality control, including guided practice abstraction and independent abstraction with reconciliation by a trainer. A 10% random sample will be dual abstracted. A RA assignment will monitor and reconcile case report forms with RedCap data once monthly.

15. Confidentiality[34]

15.1. Procedures to secure the data and/or biospecimens during storage, use, and transmission:

All medical record, health care utilization, survey data, and audio transcriptions will be coded and labeled with study identification numbers. All will be stored in original (hard copy) forms in locked cabinets (where relevant) and/or password-protected secure databases (for all electronic survey data). A1c data will be entered into the medical record (SCH) or sent to individual providers (BCM/TCH) and stored in compliance with each institutition's laboratory's protocols and procedures. At SCH, samples for HbA1c (DBS) are stored for 1-2 months at -20 C. The SCH lab keeps current and previous month samples, and samples are discarded at the first of the month. The storage locations are in the clinical lab in OC.10. Badge access is required to access this floor. At BCM/TCH, samples will be discarded seven days after receipt and processing. Storage location is WT.BB1.100 and badge access is required for this space. Original surveys will be saved for 10 years or until final analyses are completed, whichever occurs last, in order to ensure data quality. No identifiable patient information will be labeled on the surveys; all will be identified with a study-specific identifier with assigned identifier kept on a password protected encrypted server. Coded electronically saved study data will be banked indefinitely for future use by the group of investigators conducting the study and access will be controlled by the PI's. PIs will verify that additional analyses have IRB approval. Only study staff with human subjects training will have access, subject to approval by the PI. No data will be withdrawn from the study database. Results of initial analyses will be shared with participants if participants request on the consent form that they would like a summary of study results. When using web-based video chats for participant interaction, we will not store patient data/PHI within the web based applications.

Data will be stored in an electronic database (REDCap -Research Electronic Data Capture) using the participant's study identifier. REDCap data collection projects rely on

a thorough study-specific data dictionary defined as an iterative self-documenting process by all members of the research team. We have conducted the iterative development and testing process previously, resulting in well-planned data collection. REDCap servers are housed in a data center and all web-based information transmission is encrypted. For the present study, we will use the REDCap system managed by the University of Washington Institute for Translational Health Sciences (ITHS). REDCap was developed specifically around HIPAA-Security guidelines and is recommended to researchers at various institutions by both Privacy Officers and Institutional Review Boards. REDCap has been disseminated for use locally and at other institutions and currently supports over 300 academic/non-profit consortium partners on six continents and over 20,000 research end-users (www.project-redcap.org). The REDCap data record may contain some identifying information. Subjects will be tracked using their study identifier. The identifiable data will be designated as "PHI" in the REDCap database which will allow us to exclude access to the identifiable information as necessary.

When Zoom is used, the following actions will be taken to protect confidentiality and privacy:

1. The latest version of Zoom that is available will be used.
2. The meeting room will be set to private.
3. A password/passcode will be required for meeting entry.

Note: The chat function will be utilized (as needed) for individual 1:1 communication between coach and participant, including sharing approved resources (e.g., PRISM cheatsheets), logistical information about PRISM (e.g., PRISM app code), and communicating if there are technical difficulties. Chat may also be considered for use for session content by patient request to accommodate specific needs (e.g., patient is unable to communicate verbally). No PHI would be communicated via chat at any time.

15.2. Location where the data and/or biospecimens will be stored:

All will be stored in original (hard copy) forms in locked cabinets (at local sites) and/or password-protected secure databases (for all electronic survey data). A1c data will be stored in compliance with each institution's CLIA-certified laboratory policies and procedures.

15.3. Length of time data and/or biospecimens will be stored:

10 years or until final analyses are completed, whichever occurs last.

15.4. Individuals with access to data and/or biospecimens:

Only members of the study-team will have access.

15.5. Process for the transmission of data and/or biospecimens outside Seattle Children's:

15.5.1. List of data and/or biospecimens that will be transmitted:

Site RAs will maintain original copies of all consent forms and fax/email copies to the coordinating center for secure storage and tracking purposes. Upon study

completion (last data collection for last patient), all materials will be coded (labeled only with study id) and faxed or scanned to the coordinating center for electronic storage.

15.5.2. Individual(s) who will transmit data:

Site RAs, study staff, and the principal investigator/site PIs

16. Provisions to Monitor Data to Ensure the Safety of Subjects[35]

16.1. Plan to periodically evaluate the data collected regarding both harms and benefits to determine whether subjects remain safe:[36]

This is a small multisite clinical trial of a supportive care intervention that presents no more than minimal risk. As such, data monitoring will be primarily carried out by the Lead RA and a small Data Safety Monitoring Committee (DSMC).

Safety monitoring will be the responsibility of a 3-member Data Safety and Monitoring Committee (DSMC) composed of professionals representing different disciplines and expertise (see table below). All members are independent of the protocol. The committee will be convened at the beginning of the study and then twice annually via conference calls, to provide input and guidance on the study evaluation and intervention protocols, including quality assurance and safety issues related to the protocols, as well as data handling activities. As above, in the event of an unanticipated patient death, the committee may convene an ad-hoc session and/or suspend the study to assess patient risk and/or necessary revisions to the protocol.

Data and Safety Monitoring Board

Member Name, Title	Discipline	Research Expertise
David Barker, PhD (Assistant Professor, Alpert Medical School, Brown University)	Pediatric Psychologist and Applied Statistician	Pediatric Psychology, Health Behaviors
Lauren Wisk, PhD (Assistant Professor,University of California Los Angeles)	Adolescent Medicine	Pediatric Psychosocial and Health Services
Elizabeth Pyatak, PhD, OTR/L, CDE (Assistant Professor, USC)	Psychology and Behavioral Health	Diabetes Behavioral Research Interventions

16.2. Data reviewed to ensure safety of subjects:

DSMC members will provide input and feedback to the PI and Co-investigators, via e-mail and conference calls, related to (a) accrual rate, (b) study eligibility determination issues, (c) data completion rates including conformance with informed consent requirements, (d) intervention fidelity indicators, (e) adverse events, and (f) compliance with data management procedures. Dr. Zhou (lead statistician) will oversee the summarization of online data to evaluate data completeness and protocol adherence. The Lead CRA will also send semi-annual reports summarizing recruitment and other site specific data (such as indicators of intervention delivery, occurrence of adverse events, and conformance with IRB requirements).

The committee will also receive information on questionnaire data, presented for the participants overall rather than by study group. This study will not have pre-set stopping rules, but the DSMB will have the option of requesting the data be unblinded and considering altering the study or

stopping the study early. Although the full committee will meet twice-yearly, the PI or any member of the DSMB will be free to request to assemble the full committee at any time. Because committee members may be located at various sites, the committee meetings may be conducted by phone.

Data safety monitoring for the intervention will focus on assuring that subjects are not experiencing any significant or unexpected distress and that they are satisfied with the intervention components; we will monitor all complaints about the study. We do not anticipate stopping the study early for efficacy or harm, but the DSMB will have the option to consider such action in the event of a highly unexpected result. The DSMB will review the draft questionnaires to be sent to subjects and review any complaints that may be received from patients, family members, clinicians, or others. We do not anticipate any external factors such as findings from ongoing trials that will affect the safety of participants or the ethics of this research study.

16.3. Safety information collection procedures:

The DSMB, Lead RA and study team will regularly assess confidentiality of study participants. The following will be assessed and verified: All data will be maintained on secure computers in locked offices at Seattle Children's Research Institute (data collection center); the participant (and caregiver) has signed informed consent and HIPAA forms; all standard confidentiality/privacy rules and guidelines set by the Seattle Children's Research Institute are in compliance. A Certificate of Confidentiality will be obtained.

16.4. Frequency of cumulative data review:

The committee will be convened at the beginning of the study and then twice annually via conference calls, to provide input and guidance on the study evaluation and intervention protocols, including quality assurance and safety issues related to the protocols, as well as data handling activities.

16.5. Conditions that trigger an immediate suspension of the research:

Given the non-invasive nature of our proposed behavioral intervention and based on prior pilot studies,[30,32] we expect minimal side effects and safety concerns as a result of the PRISM intervention. Thus, there are no pre-planned stopping rules for adverse events since these are not anticipated.

17. Use of Social Media

17.1. Types of social media to be used and how:

n/a

17.2. Measures in place to protect the privacy or confidentiality of subjects:[37]

n/a

17.3. Types of communications that will be submitted to the IRB for review:[38]

n/a

17.4. If user-generated content will be active, how it will be monitored and what actions will be taken to ensure subject safety and study integrity:

n/a

18. Research Related Injury[39]

18.1. Available compensation in the event of research related injury:

There is no compensation for research-related injury.

19. Recruitment Methods[40]

19.1. When, where, and how potential subjects will be recruited:

Recruitment and retention will be promoted through a combination of clinic-based approaches and strategies we have successfully used previously.

Research staff at both sites will screen patients via review of clinic schedules and existing databases as well as via communication with clinicians, followed by a medical chart review to verify all eligibility except for diabetes distress to inform the in-clinic recruitment. At SCH, diabetes distress is typically screened at every in-person visit (standard of care) for all patients 13+ using the PAID-T. Clinic infrastructure provides instantaneous e-mail alerts to providers upon elevated scores. Our research team will be included in the e-mail notification of all patients with elevated scores (≥30 per published guidelines and mean clinic data).[7,52] Research staff will screen these patients for other eligibility criteria and if eligible, will work with the provider team in clinic (as available) or contact the family by phone/video conference call (WebEx, Zoom, or Skype) to conduct a consent conference if the patient/family is interested.

Both sites may also use a mail approach to aid in in-clinic screening and recruitment. Upon reviewing clinic schedules and databases and screening patients for eligibility on criteria noted in the medical record or clinic databases (e.g., age, diagnosis, duration), patients will be mailed a letter in advance of their clinic visit or sent a letter via myChart in EPIC introducing them to the study and giving them the opportunity to "opt out" of future contacts if desired. If potentially eligible patients who receive the mailing do not opt-out of being approached about the study, we may call them to assess their interest and arrange to discuss the study by phone/video or meet them in-person at their upcoming clinic visit.

Both sites may post the approved flyer in clinic areas. The flyer may also be distributed to potential participants from clinical or research staff via myChart in EPIC or by e-mail if patients and families request more information.

At SCH, the screening visit will include verification of the PAID-T measure, and if all criteria are met, the full consent conference will take place (i.e., Phase 2 of Consenting; see Figure 2 below). At BCM and SCH, if a PAID-T is not available in the medical record, the screening visit will include verification of all criteria except for the PAID-T. If the patient is approached in clinic or by phone/video and expresses interest in participating in the study, the screening consent conference will take place (i.e., Phase 1 of Consenting; see Figure 2 in Consent/Assent Section below). If the patient is available in clinic, research staff will obtain written consent/assent for Phase 1/Screening prior to administering the PAID-T to the adolescent. If the patient is not in

clinic and research staff is following up by phone/video, verbal consent/assent for Phase 1/Screening will be obtained via the information sheet prior to PAID-T administration. The patient will only be eligible for Phase 2 of Consenting and to be enrolled if the PAID-T is elevated (>=30). The same two-phase consenting process will occur at SCH if a PAID-T is unavailable at the time of consent (e.g., patient has opted out of taking standard of care measures in clinic, has not received the PAID-T as part of a tele-health visit or in-person visit or contacts the study team after receiving the recruitment letter but does not have a PAID-T in chart within the prior 12 months). In cases where the PAID-T is elevated upon screening and the patient/family subsequently decides not to participate, the provider will be notified (if not already notified).

19.2. Steps that will be taken to protect potential subjects' privacy interests:[41]

A variety of measures will be utilized to ensure participant privacy. We will follow standard processes for approach by using letters and/or working with the diabetes provider team to approach in person. Minimal paper records, such as consent forms, will be kept in a locked drawer in the site PIs' research offices.Surveys will be done either electronically via RedCap or mailed to participants based on their preferences. A1c at-home test kits will follow clinic and laboratory protocols as defined by each institution and overseen by laboratory directors at each respective institution (Dickerson & Devaraj).

19.3. Sources of subjects:[42]

We will recruit from the diabetes clinics at Seattle Children's Hospital and Texas Children's Hospital.

19.4. Methods that will be used to identify potential subjects:

RAs at each site will screen patients via review of the clinic lists, communication with clinicians, and review of internal databases, followed by a medical chart review to verify eligibility.

19.5. Materials that will be used to recruit subjects:[43]

Recruitment letter and flyer are included in the submission materials.

19.6. Recruitment methods not controlled by Seattle Children's:

There are no study wide recruitment methods (e.g., call centers or national advertisements).

20. Consent/Assent Process

20.1. Consent process overview:[44]

RAs will conduct in-person recruitment in clinic waiting rooms, at clinic visits and/or inpatient hospital rooms. Consent discussions may also occur by phone/video. Phone/video (via

WebEx, Zoom, or Skype) consents will be used in instances where patients are coming to main hospital clinics infrequently (e.g., every 6 months; not coming in for clinic visits due to COVID-19 clinic restrictions). In sum, allowing phone/video consents is necessary for maximizing recruitment and accommodating all diabetes patients, especially in the era of the COVID-19 pandemic. Some patients come to clinic once every 3-6 months, and clinic visits have become less frequent due to increased infection risk as a result of COVID-19. In these instances and in the current circumstances, it is sometimes impossible to ensure that an in person consent conference occurs if a study team member is not allowed to be in clinic due to institutional safety protocols (e.g., COVID-19 restructions), out for the day or if our team has a scheduled meeting. Even if a study team member is able to be at the hospital, there are a number of factors that may preclude an approach in person, especially in the era of COVID-19. Providers may interrupt the consent conference, staff may advise us that the day is not “good” for the family to be approached, illness status may preclude the patient from being cognitively able to provide consent/assent that day, or patients/families may not be presenting in person in clinic due to COVID-19.

The RA will contact families and ask if they are interested in learning about a research study. The consent meeting between the RA and eligible participants (with parents if applicable) will include an explanation of the study in developmentally appropriate lay-language.

For Phase 1 (Screening), participants will provide either written informed consent (if aged 18 years) or assent (if <18 years-old) if seen in-person or verbal consent (if aged 18 years) or assent (if <18 years-old) if screened by phone/video. Parents of teens (<18 years-old) will provide written informed consent if the patient is seen in clinic or verbal consent if screened by phone/video.

For Phase 2 (Full Study), all participants approached in-person will provide either written informed consent (if aged 18 years) or assent (if <18 years-old). Parents of teens (<18 years-old) approached in-person will provide written informed consent. When the consent conference is conducted by phone/video, those who agree to participate will complete an electronic consent form via the REDcap database using the REDCAP e-consent framework. Study staff may also e-mail a copy of the consent form to subjects if requested. The body of the consent form will be identical, whether on paper or in REDCap. A link to the electronic consent form will be e-mailed to participants prior to the phone/video consent conference. A short code will be generated through REDCap to confirm and verify the identity of the person consenting at BCM/TCH. Study staff will answer any questions about the study, and those who agree to participate will provide an electronic signature, date, and time on the REDCap form to indicate their consent. The REDCap e-consent framework allows participants to create an electronic signature using their cursor and provides a timestamp. This framework also automatically generates an extra certification page for participants to confirm the correctness of their responses before submitting and stores a static copy of their responses as a PDF in the study file’s repository. Subjects will also be prompted to download a copy of their signed consent form from REDCap or study staff can e-mail the signed form.

After conducting the consent conference either in person or by phone/video, RAs will create a consent process note for REDCap and the EMR. Included in this note will be the date of

the consent conference, the names of the patient and parent (if applicable), the name of the person obtaining consent, the fact that the subject agreed to participate and that all risks and opportunities of the study were explained, and that the subject had adequate opportunity to have their questions answered. If an interpreter is used, the name of the interpreter will be documented.

Consent conferences will not be recorded. Study staff will use a guide for consent discussions over phone/video.

After fully enrolled participants turn 18, they will complete a new informed consent form to continue participation in the study either in-person (as is possible) or via REDCap.

As described in the inclusion/exclusion criteria above, adolescents must speak English fluently; however, they are eligible if English is not their primary language spoken at home (or if it is not their first language). In cases where parent consent is required and parents do not speak English fluently (or prefer another language), all conferences will be held with a trained phone or in-person medical interpreter. All participants and parents will be provided an opportunity to read the consent/assent forms in their preferred language (English or Spanish), to ask questions about the study and have those questions answered by the research team member before deciding about study participation and signing the consent/assent form. Parental permission for study participation will be obtained first then patient assent will be obtained for all patients 13-17 years of age. If a patient indicates that they do not want to participate in any phase of the study that non-assent will override the parent's permission and the patient will be recorded as a refusal. The research team member will redirect any parent who attempts to convince their child to participate in the study and remind them of their child's right as a potential research participant to refuse participation without coercion. Consent to study participation will be obtained from patients 18 years of age and above. The RA will emphasize to all patients and parents in developmentally appropriate lay-language that being in the study is their choice that they may choose not to participate or may change their mind at any time and it will not affect how their nurses or doctors care for them.

Parent/guardian separate consent will be obtained because parents will be completing their own study activities and will be disclosing their own financial information in surveys. At the time of consent, the study activities associated with each arm of the study (surveys for both groups and 'Coming Together' for parents/guardians whose child is randomized to PRISM) will be presented, and parents can indicate on the consent which component(s) they are interested in participating in (with the knowledge that they will not be offered the 'Coming Together' session should their child be randomized to control/usual care). Again, parents/guardians whose child is randomized to control/usual care arm will be eligible to take surveys but will not have the option of the 'Coming Together' visit (as it is a part of the PRISM intervention). Parents/guardians whose child is randomized to the PRISM arm of the intervention may choose to be involved in the 'Coming Together' portion of the study and not want to complete questionnaires and visa versa, they may choose to complete questionnaires and not participate in the 'Coming Together' portion of the study either by their or the patient's choice. It is clearly delineated on the parent consent forms both a) that the parent should indicate which component(s) they are choosing to participate in (if eligible)

and b) that the ‘Coming Together’ session is only offered if the adolescent is randomized to PRISM.

Screening Procedures (Phase 1):

During each consent conference at BCM/TCH, where distress screening (PAID-T) is not standard of care, and for consent conferences at SCH in which the patient has not been administered a PAID-T, we will emphasize that they will need to complete a screening consent form or information sheet (Phase 1) and a brief measure (PAID-T) in order to determine eligibility (see Figure 2 below). If they meet eligibility criteria, they will proceed to Phase 2 of Consenting and will subsequently complete the baseline instruments and will not be given the PAID-T again (i.e., the PAID-T score from screening will count as their baseline PAID-T value). If they do not meet eligibility criteria, we will thank them for their time and confirm they are not eligible for the study. If they are eligible and interested in the study, we will ask them to complete the full consenting process (Phase 2; see Figure 2 below) and the rest of the baseline surveys prior to being randomized. The same processes will occur at SCH if the potentially eligible patient does not have a current PAID-T available in their chart (i.e., within 12 months of the time of consent).

All potential participants who complete the PAID-T as part of the Phase 1 screening process (outside of their standard of care PAID-T) with the intention of enrolling in the study who do not subsequently meet the cut-off for enrollment will be given a $5 gift card for their time. They will complete the PAID-T in the run in period via REDCap, verbally, or paper/pencil.

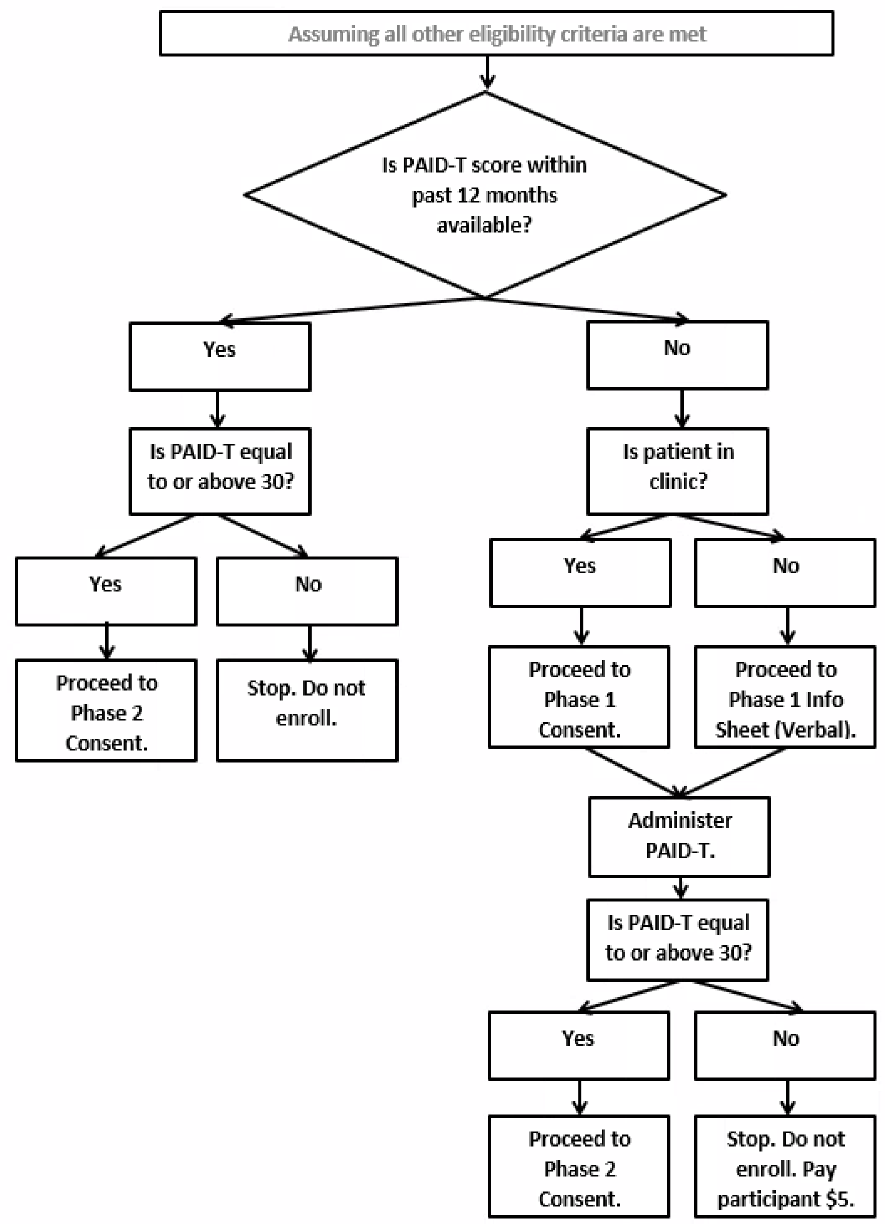

Figure 2. Two-phase consenting flow chart.

20.2. Where the consent process will take place:

In the diabetes clinic setting at both sites, the Clinical Research Center, or by phone/video.

20.3. Steps that will be taken to protect prospective subjects' privacy interests:[45]

The study team will use a private room or private part of the waiting room to discuss potential participation and the use of an intermediary (as needed) if the subject does not know the researcher. If consenting by phone/video, we will request that potential participants find a quiet, private location to meet. Emphasis will be made to ensure that subjects know that not participating will not impact patient care.

The study team will warn the participant of the possibility of sensitive subject matter before the PRISM session (if applicable per randomization). The study team will be sure to emphasize to the participant that this study is completely optional and the participant has the right to not answer any questions that they feel uncomfortable with, that they can withdraw their participation at any time, and that their refusal to participate in this research will not impact their care.

20.4. Waiting period available between approaching a prospective subject and obtaining consent:
n/a – they can consent at time of approach.

20.5. Process to ensure ongoing consent:
If a minor patient turns 18 while still on study, we will reconsent them as an adult. We will also continue to emphasize that research participation is optional and does not affect care at SCH/BCM throughout the study.

20.6. If this box is checked, "SOP: Informed Consent Process for Research (HRP-090)" will be followed: ☒

20.7. If "SOP: Informed Consent Process for Research (HRP-090)" will not be followed, address the following:[46]

20.7.1. Role of the individuals listed in the application as being involved in the consent process:
✍Click here to enter text.

20.7.2. Time that will be devoted to the consent discussion:
✍Click here to enter text.

20.7.3. Steps that will be taken to minimize the possibility of coercion or undue influence:
✍Click here to enter text.

20.7.4. Steps that will be taken to ensure the subject's understanding:
✍Click here to enter text.

20.8. Non-English Speaking Subjects[47]

20.8.1. Anticipated preferred language(s) for subjects or their representatives:
English/Spanish

20.8.2. Presentation of Research Information and Documentation:

☐ Appendix A-10 of the Investigator Manual will be followed[48]

☐ Short form procedures may be used per HRP-091. If so, choose applicable box(es):

☐ Per section 5.5.1

☐ Per section 5.5.2

☒ Appendix A-10 of the Investigator Manual will not be followed. Explanation of procedures not following Appendix A-10:

Although Spanish speaking families will be eligible for the study only if the adolescent can speak fluently in English, adolescents who do not speak English will be ineligible for the study as PRISM is a manualized intervention only available and iteratively developed in English. In cases where parent consent is required and parents do not speak English fluently (or prefer another language), all conferences will be done with a trained medical interpreter. All participants and parents will be provided an opportunity to read the consent/assent form in their preferred language (English or Spanish), to ask questions about the study and have those questions answered by the research team member before deciding about study participation and signing the consent/assent form. All surveys and consent documents for parents will also be available in Spanish.

20.8.3. Justification if non-English speaking subjects will be excluded from the research:[49]

The PRISM intervention and materials are not manualized in non-English languages and were not iterively developed through rigorous feasibility testing in any other language.

20.9. Subjects Who Are Not Yet Adults (Infants, Children, Teenagers)

20.9.1. Process used to determine whether an individual has not attained the legal age of consent under the applicable law of the jurisdiction in which the research will be conducted (e.g., individuals under the age of 18 years):[50]

Age will be verified via medical record.

20.9.2. Parental permission will be obtained from:[51]

☐ Both parents unless one parent is deceased, unknown, incompetent, or not reasonably available, or when only one parent has legal responsibility for the care and custody of the child.

☒ One parent even if the other parent is alive, known, competent, reasonably available, and shares legal responsibility for the care and custody of the child.

☐ Neither parent.[52]

20.9.3. Process used to determine an individual's authority to consent to each child's general medical care if permission will be obtained from someone other than parents:[53]

The medical record indicates legal guardianship (i.e., who can give consent for their child to be enrolled on research studies). As such, we will only obtain consent for AYAs under 18 when a legal guardian, as indicated by the medical record, is present for the consent conference.

20.9.4. Assent will be obtained from:[54]

☒ All children.
☐ Some children. Specify: ✍Click here to enter text.
☐ None of the children. Explain: ✍Click here to enter text.

20.9.5. Procedures for obtaining and documenting assent:

Phase 1 (Screening): Written assent will be obtained for patients 13-17 (Screening Consent/Assent/Parental Permission), unless it is not possible for research staff to meet patient/family in person. If patient/family is not able to be seen in person, verbal assent will be obtained (Information Sheet).

Phase 2 (Full Consent): Written assent will be obtained for all patients 13-17 if approached in clinic. REDCap e-assent will be obtained for all patients who are approached by phone/video These consent conferences will be documented in the patient's medical record. A copy of the consent/assent/parental permission form will be e-mailed/mailed prior to the phone/video consent conference, upon request.

If a patient indicates that he or she does not want to participate in the study during any phase, that non-assent will override the parent's permission and the parent will be recorded as a refusal. The research team member will redirect any parent who attempts to convince their child to participate in the study and remind them of their child's right as a potential research participant to refuse participation without coercion. The CRA or PI will emphasize to all patients and parents in developmentally appropriate lay-language that being in the study is their choice, that they may choose not to participate or may change their mind at any time and it will not affect how their nurses or doctors care for them.

20.9.6. Plan for re-approaching children who have reached the age of majority to obtain consent:[55]

We will re-approach and consent the children of participants if they turn 18 while they are still actively participating in Phase 2 of the study. Reapproach will be conducted after the participant turns 18 and before completing surveys or interventions. However, we request the waiver of consent for patients that turn 18 if no further data collection and study related activities are occurring, their data is limited to the continued use of existing data.

20.10. Cognitively Impaired Adults/Adults Unable to Consent[56]

20.10.1. Process used to determine whether an individual is capable of consent:

When determining eligibility with a medical record screening, we will look for any indication of inability to provide assent/consent (i.e., medical conditions, cognitive conditions, etc.). If there are any indicators of potential inability to

provide assent/consent, the RA will reach out to the PI and the patient's medical provider to determine eligibility to provide assent/consent.

20.10.2. Individuals from whom permission will be obtained in order of priority:[57]
N/A

20.10.3. Assent will be obtained from:
☐ All of these subjects.
☐ Some of these subjects. Specify: ✍Click here to enter text.
☐ None of these subjects. Explain: ✍Click here to enter text.

20.10.4. Process for obtaining and documenting assent:[58]
Patients unable to provide assent/consent will not be eligible for this study. Please see section 20.9.5 for processes and procedures for obtaining assent from eligible patients.

20.11. Waiver or Alteration of Consent Process

20.11.1. Reasons for requesting a waiver or alteration of informed consent:[59]

1) We are requesting a waiver of consent for patients that turn 18 if no further data collection and study related activities are occurring (see Section 20.9.6) as their data is limited to the continued use of existing data. It would not be practical to seek consent after study activities are complete because of the potential difficulty locating subjects. It may be inappropriate or unnecessarily burdensome to patients to re-approach them after study activities are complete.

20.11.2. Consent Waiver/Alteration Criteria justifications:[60]

20.11.2.1. The research involves no more than minimal risk to the subjects because: The waiver includes continued use of existing data.

20.11.2.2. The waiver or alteration will not adversely affect the rights or welfare of the subjects because:[61] Participants assented to participate in the study. All publication or presentation of research results will be done in a manner that would not reveal an individual's identity.

20.11.2.3. The research could not practicably be carried out without the waiver or alteration because:[62]

- We may be unable to locate patients because of the lengthy time period over which the records will be collected
- All eligible patients (especially those who have completed study activities) must be included in the study for the results to be meaningful

If the research involves using identifiable private information or identifiable biospecimens, the research could not practicably be carried out without using such information or biospecimens in an identifiable format because: Identifiers are necessary so that researchers can link data from multiple sources for data analysis.

20.11.2.4. Whenever appropriate, the subjects will be provided with additional pertinent information after participation:

If indicated by participants, a summary of the primary analyses will be shared at the study closure.

20.11.3. If the research involves a waiver of the consent process for emergency research, provide sufficient information for the IRB to make it determinations:[63]

n/a

21. Process to Document Consent in Writing

21.1. If consent will be documented in writing (check one):

☒ "SOP: Written Documentation of Consent (HRP-091)" will be followed.

☒ "SOP: Written Documentation of Consent (HRP-091)" will not be followed. Process of documenting consent:[64]

HRP-091 will be followed for Phase 1 (Screening) if patient/family are available in clinic to provide signatures. Due to the relative infrequency of clinic visits, for instances in which the patient/family are NOT available to meet in-person, we are requesting an alteration of consent, which allows the patient/family to verbally consent and assent (e.g., by telephone/video) to the screening procedures in Phase 1.

HRP-091 will be followed for Phase 2 (Full Study) when patient can be approached in person. We will obtain written assent/consent from all participants. Upon enrollment and randomization in Phase 2, the PI or Co-Is will place a research note in the medical record to document the discussion and participants. HRP-091 will not be followed for Phase 2 (Full Study) when patient is approached remotely. We are requesting a waiver of documentation of written consent when approach and consenting are performed via telephone or video conference. Identical REDCap consent forms are drafted for remote consenting, which include signature fields for assent and consent.

We will obtain written assent/consent from participants whenever possible as a wet ink of signature. When this is not possible, we will obtain a digital signature to indicate willingness to participate.

Consent documents, including the identical REDCap consent forms for remote consenting, are attached in the appropriate section of the Click smart form.

21.2. If consent will not be documented in writing (check all boxes that apply):[65]

- ☒ A written statement/information sheet describing the research will be provided to subjects.[66]
- ☐ A written statement/information sheet describing the research will not be provided to subjects. Explain: ✍Click here to enter text.
- ☒ A consent script will be used.[67]

22. HIPAA Authorization and RCW Criteria

22.1. HIPAA Authorization (check all boxes that apply):

- ☐ The study does not involve the receipt, creation, use and/or disclosure of protected health information (PHI).[68]
- ☒ HIPAA authorization will be obtained as part of a signed consent form.
- ☒ The study will access PHI without prior authorization from subjects (including for recruitment purposes – e.g., reviewing the medical record to determine eligibility). *See 21.2 below for required HIPAA waiver/alteration criteria*.
- ☒ Subjects will review a written statement/information sheet with the appropriate HIPAA language but will not provide a written signature. See 21.2 below for required *HIPAA alteration criteria*.[69]
- ☐ Other. Explain:[70]
  ✍Click here to enter text.

22.2. HIPAA Waiver/Alteration Criteria: Explain why:

22.2.1. The use or disclosure of PHI involves no more than a minimal risk to privacy of individuals, based on, at least the presence of the following elements:

22.2.1.1. An adequate plan to protect the identifiers from improper use and disclosure:

Access PHI for Screening: All screening will take place using secure login credentials and databases/medical records limited to access only by study staff. All study staff and investigators are highly trained in HIPAA compliance, human subjects protection, and good clinical practice.

Remove Signature Requirement for Phase 1 Screening and Phase 2 Consent (if not seen in-person): We are requesting an alteration of HIPAA authorization to remove the signature requirement for Phase 1 Screening if screening is done by phone (and a signature cannot be obtained in-person) and for Phase 2 Enrollment if done by phone/video (and a signature cannot be obtained in person). All study materials will be filed in locked cabinets and study staff and investigators are highly trained in HIPAA compliance, human subjects protection, and good clinical practice.

22.2.1.2. An adequate plan to destroy identifiers at earliest opportunity consistent with conduct of research:

Access PHI For Screening: All information collected for research purposes will be coded. Identifying information (names, addresses

and phone numbers) will be used initially only to identify potential patients to approach. The only link between the participant identifiers and their study identifier will be kept on a password protected database and in a locked filing cabinet. There are no patient identifiers collected and retained for research purposes. Feasibility data will represent only frequencies and percentages.

Any remaining identified information (i.e., databases) will be destroyed at 10 years or after final analyses are completed, whichever occurs last, in order to ensure data quality.

Remove Signature Requirement for Phase 1 Screening and Phase 2 Enrollment (if not seen in person): All information collected will be coded. The only links between the participant identifiers and their study identifiers will be kept on a password protected database and in a locked filing cabinet. There are no patient identifiers collected and retained for research purposes.

22.2.1.3. Assurances that PHI will not be reused or disclosed to any other party or entity, except as required by law or for authorized oversight of the research:

Access PHI for Screening: PHI will not be reused or disclosed to any other party or entity, except as required by law or for authorized oversight of research.

Remove Signature Requirement for Phase 1 Screening and Phase 2 Enrollment (if not seen in person): PHI will not be reused or disclosed to any other party or entity, except as required by law or for authorized oversight of research.

22.2.2. The research could not practicably be conducted without the waiver or alteration of authorization:

Access PHI for Screening: We need to have the alteration in order to determine potentially eligible participants for this study. If we did not confirm eligibility with the clinicians giving care, confirmation of eligibility would be dependent upon the patient and parents. It is likely that they would be able to confirm the eligibility criteria but there is the possibility that some may not be able to do so for all of the study criteria. It is also possible that they may be uncomfortable being asked to confirm the eligibility criteria or wonder why it is that they are being asked to do so.

Remove Signature Requirement for Phase 1 Screening and Phase 2 Enrollment (if not seen in person): Due to the relative infrequency of clinic visits, it may not be possible to screen all potentially eligible patients in person. If the signature requirement

was not removed for potentially eligible parties, it would not be practicable to be able to screen these patients appropriately. Patients may be interested in participating in the study, and it is not reasonable to ask interested parties to wait months until their next clinic visit to engage in minimal risk, brief screening processes (<5 minutes) to assess eligbility.

- We also need this alteration to be able to recruit participants in cases where in-person consent conferences may pose risk to the patients, particularly within this immunosuppressed, higher-risk population. For example, as of Spring 2020, the COVID-19 pandemic has significantly impacted our ability to approach patients and conduct consent conferences in person; therefore, this has led us to revisit our previous practices for in-person consenting and determine that, in order to protect patient safety while still giving all eligible patients the opportunity to participate in this potentially beneficial research, we need the options to enroll patients remotely.
- In addition, both of our previous procedures for remote consent conferences are no longer feasible in this circumstance, as follows: a) when a remote consent conference was requested by patients or families, we had previously mailed consent forms prior to a phone consent conference and requested that families sign the forms and return via mail. Based on early pilot data, patients and families who were interested in the study found this to be particularly burdensome exacerbated now by families execising extreme caution to protect their child's health, meaning many may prefer to avoid sharing of physical documents via mail. Furthermore, requiring families to return documents via mail may pose additional risk and burden as they will need to leave their homes to locate mailboxes or visit the post office; b) we had previously e-mailed consent forms to families and requested that they print, sign, scan, and return to us via email. However, we have found that this is not doable for this population as it requires them to have access to a printer and scanner, which the majority do not. Therefore, in order to recruit for this study whilst ensuring patient safety and minimizing any burden or stress on families, providing an electronic means for families to review consent forms and indicate willingness to participate (via digital signature) is necessary.

22.2.3. The research could not practicably be conducted without access to and use of the PHI:[71]

Access PHI for Screening: The alteration of HIPAA Authorization is being requested for preliminary screening purposes only. Preliminary screening procedures are minimal risk; they include a basic review of the patient's medical records to identify whether or not the patient meet basic eligibility requirements. No study activities occur prior to the documentation of informed consent/authorization by patient and/or parent participants.

Remove Signature Requirement for Phase 1 Screening and Phase 2 Enrollment (if not seen in person): The nature of the research is specific to individuals' health and requires access to individuals' health records.

23. Payments/Costs to Subjects[72]

23.1. Amount, method, and timing of payments to subjects:[73]

Patients in both arms will receive a $20 gift card upon completion of baseline surveys, and a $30 gift card following the completion of the 6-month and also 12-month surveys. Because the 3-month and 9-month surveys are abbreviated, patients will receive a $10 gift card for those. At SCH, Clincards will be used for gift card distribution. At BCM/TCH, Clincards will we used for gift card distribution, unless they are not available, in which case, cash or check will be used. Parents will not be compensated. Subjects will not be compensated for participating in any intervention sessions, including the Coming Together session.

In addition, all participants who contact the research team and "opt in" via the recruitment letter, resulting in a screening visit and/or a chart review will be entered into a lottery to win a bonus $50 gift card. We will complete the lottery every 3 months or each time after 20 families enroll, whichever occurs first. We will use existing clinic data to mail letters to participants who have met inclusion criteria in the past. This is a process that has worked well for other researchers
in the diabetes clinic.

Finally, if the patient completes Phase 1 but does not qualify for the study due to the PAID-T score not being elevated, they will be offered a $5 gift card as a thank you. Please note that Phase 1 will be somewhat rare at SCH because the PAID-T and A1C are standard of care. Phase 1 screening for elevated PAID-T will occur for every participant at BCM/TCH because the PAID-T is not standard of care (A1C draws are standard of care at BCM/TCH). Those who do not qualify for the study based on their PAID-T scores or are eligible, but do not want to participate in Phase 2 will receive the $5 incentive for completing the screener. Those who do qualify for the study based on their PAID-T scores and decide to enroll in Phase 2 will not receive the $5 incentive because they will receive the full $20 incentive for completing baseline questionnaires.

Subjects who leave the study early and are asked to complete the abbreviated surveys would not be paid for the early terminiation surveys.

23.2. Reimbursement provided to subjects:**[74]**

n/a

23.3. Additional costs that subjects may be responsible for because of participation in the research:**[75]**

n/a

24. Setting

24.1. Site(s) or location(s) where the research team will conduct the research:

Diabetes clinics at Seattle Children's and Texas Children's Hospitals. Study visits may be done in person at the hospital/clinic, in the inpatient room or in the outpatient clinical research center. PRISM sessions may also be conducted by phone or other web-based communication (Zoom, Webex, or Skype).

24.2. Composition and involvement of any community advisory board:

n/a

24.3. For research conducted outside of the organization and its affiliates:**[76]**

24.3.1. Site-specific regulations or customs affecting the research:

See HRP-815 Basic Site Information for Baylor College of Medicine's Local context

24.3.2. Local scientific and ethical review structure:

n/a – SCH will be the sIRB.

25. Resources Available

25.1. Qualifications (e.g., training, education, experience, oversight) of investigator(s) to conduct and supervise the research:**[77]**

This investigative team has the qualifications and collaborative experience to complete a RCT to evaluate the clinical impact and value of a psychosocial intervention for adolescents with T1D. PI Dr. Joyce Yi-Frazier is a research psychologist with over 15 years of experience in behavioral diabetes research and stress resilience.[14,49-53] With Co-I Dr. Abby Rosenberg, head of Adolescent and Young Adult (AYA) Oncology at Seattle Children's (SCH), they developed the PRISM intervention and completed feasibility trials in adolescents with diabetes, cancer, and cystic fibrosis (craniofacial and end-stage renal disease feasibility trials are in-progress), as well as in parents of adolescents with cancer and diabetes.[29,30,79] They have refined processes of intervention training, fidelity, enrollment, study implementation and data collection. Dr. Rosenberg has served as Yi-Frazier's research mentor for the last 5 years as PI of two pilot RCTs exploring PRISM efficacy in the AYA cancer population[31] and for caregivers of AYAs with cancer (in progress). Rosenberg is currently leading an R01 multi-site trial for PRISM in one AYA cancer population, with a second awaiting SRC review with a fundable score. For this

proposal, they are supported by Co-I Dr. Marisa Hilliard (Baylor College of Medicine, BCM and Texas Children's Hospital, TCH), a pediatric psychologist with extensive experience in stress, resilience, strengths-based interventions, and PROs in adolescents with diabetes.[20,80-89] Hilliard co-led the organization of the PRO conference in November 2017, which served as the basis of this RFA. BCM/TCH is also one of the sites engaged in the multisite PRISM R01 for cancer. Other key contributors include consultant Dr. Barbara Anderson (BCM/TCH), a leading behavioral scientist in pediatric diabetes with many years of experience running clinical trials,[90-93] and Drs. Cate Pihoker, Faisal Malik (SCH) and Dan DeSalvo (BCM/TCH), pediatric endocrinologists with extensive clinical and research expertise in pediatric diabetes management.[94-96] Senior biostatisticians at SCH will lead a biostatistics support team to conduct randomization, design implementation and statistical analysis as have been done for previous PRISM studies. Finally, students will be performing administrative tasks for the study.

25.2. Other resources available to conduct the research:[78]

Fidelity. PRISM has been standardized through the creation of comprehensive protocols for those who implement it. Session-by-Session details are provided in the PRISM manual; on outline of each section is listed in **Table 1.** All interventionists undergo at least 4 hours of in person training including role-playing and progressive mastery of intervention materials. The fidelity of sessions will be systematically assessed via audio-recording.

Access to Diabetes Clinics. The research team will work closely with the Diabetes Clinics to ensure screening/enrollment is feasible. Using the eligibility criteria age, A1C, and language against our existing databases at SCH, we estimate over 400 eligible adolescents per year, 40% of whom report using a CGM. At TCH, there are over 500 eligible adolescents per year, 26% of whom report using a CGM. Assuming an enrollment rate of 50% (based on pilot data), we expect it to be feasible to enroll, randomize, and conduct the intervention for at least 80 eligible adolescents at each site in 18 months. Appropriate FTE is designated in the budget for this work. We will use the Seattle Children's Hospital Main Campus Diabetes Clinic and all regional clinics to conduct this research protocol. BCM/TCH will also use their Main Campus Diabetes Clinic and regional sites.

26. Coordinating Center Procedures

26.1. Coordinating center institution:

SCH

26.2. If Seattle Children's is the coordinating center:

26.2.1. Process to ensure communication among sites:[79]

Each site will be supported by a PI, endocrinologist/diabetologist, senior consultant, and study staff including an interventionist and research associate (RA). The lead RA from both sites will have regular weekly contacts to ensure all study procedures are maintained. Specifically:

SCH will be the primary administrative/data coordinating site and provide the following additional oversight and responsibilities:

- Administrative oversight:
    - SCH staff will set-up all tracking databases.
    - SCH staff will set-up all REDCap databases.
    - SCH will oversee the budget.
- Intervention-specific oversight:
    - SCH staff will provide all training for PRISM in an in-person training session
    - Lead interventionist will monitor training, fidelity, and supervision of interventionists, including an in-house development program designed to monitor interventionist engagement and potential for burnout.
- Biostatistical oversight:
    - Randomization scheme to be created by SCH biostatisticians and disseminated to BCM/TCH via REDCap.
    - Data from BCM/TCH will be sent to SCH after baseline, 6-month, and 12-month data is collected.
    - Combined data analysis for both PRISM efficacy (Aims 1 and 2) will be conducted by SCH biostatisticians.

26.2.2. Process to ensure all site investigators conduct the study according to the IRB approved protocol and report all non-compliance:

All site investigators will conduct the study according to the IRB approved protocol and report all non-compliance. Staff at SCH will have both internal meetings to discuss and report any non-compliance and with external sites. The study will also be monitored by the DSMC. All study staff, investigators, and students will be required to review the protocol, have GCP/HSP training, and submit financial disclosures. The process for reporting reportable new information (RNI) will be reviewed with all staff and investigators, as outlined in the investigator's manual.

26.2.3. Process to ensure all required approvals are obtained at each site:

We will hold a protocol implementation meeting prior to study launch at both sites to ensure all required approvals are obtained.

26.2.4. Process to ensure all sites are informed of any problems and/or interim results:

The lead study RA and/or PI will hold monthly meetings with each site to review problems and/or interim results. Additionally, problems and interim results will be brought to the attention of the DSMC. If problems and/or interim results are urgent and require immediate review, the lead study RA and/or PI will contact BCM/TCH, the DSMC, and/or the IRB, as needed and consistent with IRB rules, federal law, and Good Clinical Practice.

27. International Center for Harmonization of Good Clinical Practice (ICH-GCP)

27.1. If you have committed to conducting the described study per ICH-GCP, check this box: ☒[80]

- This is generally applicable for contracts with industry-sponsored studies or sponsor protocols. See your contract/agreement or Sponsor Documentation if you are unsure.
- Note that completing GCP training is a separate activity and does not automatically mean that you have committed to conducting the study per ICH-GCP.
- **If you check the box, upload a current curriculum vitae (CV) for the PI to the "Other Attachments" section of the "Local Site Documents" SmartForm.**

1. Hagger V, Hendrieckx C, Sturt J, Skinner TC, Speight J. Diabetes distress among adolescents with Type 1 Diabetes: a systematic review. *Curr Diab Rep.* 2016;16:9.
2. Lašaitė L, Dobrovolskienė R, Danytė E, et al. Diabetes distress in males and females with type 1 diabetes in adolescence and emerging adulthood. *J Diabetes Complications.* 2016;30:1500-1505.
3. Fisher L, Glasgow RE, Mullan JT, Skaff MM, Polonsky WH. Development of a brief diabetes distress screening instrument. *Ann Fam Med.* 2008;6:246-252.
4. Grey M, Whittemore R, Tamborlane W. Depression in type 1 diabetes in children: natural history and correlates. *J Psychosom Res.* 2002;53:907-911.
5. Grigsby AB, Anderson RJ, Freedland KE, Clouse RE, Lustman PJ. Prevalence of anxiety in adults with diabetes: a systematic review. *J Psychosom Res.* 2002;53:1053-1060.
6. Kovacs M, Goldston D, Obrosky DS, Bonar LK. Psychiatric disorders in youths with IDDM: rates and risk factors. *Diabetes Care.* 1997;20:36-44.
7. Weissberg-Benchell J, Antisdel-Lomaglio J. Diabetes-specific emotional distress among adolescents: feasibility, reliability, and validity of the problem areas in diabetes-teen version. *Pediatric Diabetes.* 2011;12:341-344.
8. McGrady M, Laffel LM, Drotar D, Repaske DR, Hood KK. Depressive symptoms and glycemic control in adolescents with type 1 diabetes: Mediational role of blood glucose monitoring. *Diabetes Care.* 2009;32(5):804-806.
9. Hilliard ME, Yi-Frazier JP, Hessler D, Butler AM, Anderson BJ, Jaser S. Stress and A1c among people with diabetes across the lifespan. *Current Diabetes Reports.* 2016;16:67.
10. Helgeson VS, Vaughn AK, Seltman H, Orchard T, Libman I, Becker D. Trajectories of glycemic control over adolescence and emerging adulthood: An 11-Year longitudinal study of youth with Type 1 Diabetes. *J Pediatr Psychol.* 2018;43:8-18.
11. Hagger V, Hendrieckx C, Cameron F, Pouwer F, Skinner TC, Speight J. Diabetes distress is more strongly associated with HbA1c than depressive symptoms in adolescents with type 1 diabetes: Results from Diabetes MILES Youth-Australia. *Pediatr Diabetes.* 2018;epub ahead of print.
12. Hale DR, Viner RM. The correlates and course of multiple health risk behaviour in adolescence. *BMC Public Health.* 2016;31:458.
13. Kipping RR, Campbell RM, MacArthur GJ, Gunnell DJ, Hickman M. Multiple risk behaviour in adolescence. *J Public Health.* 2012;34:Suppl1: i1-2.
14. Delamater AM, de Wit M, McDarby V, Malik J, Acerini CL, International Society for Pediatric and Adolescent Diabetes. ISPAD Clinical Practice Consensus Guidelines 2014. Psychological care of children and adolescents with type 1 diabetes. *Pediatr Diabetes.* 2014;15(Suppl 20):232-244.
15. Chiang JL, Kirkman MS, Laffel LM, Peters AL, Type 1 Diabetes Sourcebook Authors. Type 1 diabetes through the life span: a position statement of the American Diabetes Association. *Diabetes Care.* 2014;37:2034-2054.
16. de Wit M, Pulgaron ER, Pattino-Fernandez AM, Delamater AM. Psychological support for children with diabetes: are the guidelines being met? *J Clin Psychol Med Settings.* 2014;21:190-199.
17. Attari A, Sartippour M, Amini M, Haghighi S. Effect of stress management training on glycemic control in patients with type 1 diabetes. *Diabetes Res Clin Pract.* 2006;73:23-28.
18. Hannon TS, Yazel-Smith LG, Hatton AS, et al. Advancing diabetes management in adolescents: Comparative effectiveness of mobile self-monitoring blood glucose technology and family-centered goal setting. *Pediatr Diabetes.* 2018;epub ahead of print.
19. Huston SA, Blount RL, Heidesch T, Southwood R. Resilience, emotion processing and emotion expression among youth with type 1 diabetes. *Pediatr Diabetes.* 2016;17:623-631.
20. Hilliard ME, Harris MA, Weissberg-Benchell J. Diabetes resilience: a model of risk and protection in Type 1 Diabetes. *Curr Diab Rep.* 2012;12:739-748.

21. Yi-Frazier JP, Yaptangco M, Semana S, et al. The association of personal resilience with stress, coping and diabetes outcomes in adolescents with Type 1 Diabetes: variable and person-focused approaches. *Journal of Health Psychology.* 2013;20:1196-1206.
22. Rosenberg AR, Yi-Frazier JP, Wharton C, Gordon K, Jones B. Contributors and Inhibitors of Resilience Among Adolescents and Young Adults with Cancer. *J Adolesc Young Adult Oncol.* 2014;3(4):185-193.
23. Rosenberg AR, Baker KS, Syrjala KL, Back AL, Wolfe J. Promoting resilience among parents and caregivers of children with cancer. *J Palliat Med.* 2013;16:645-352.
24. Rosenberg AR, Wolfe J, Bradford MC, et al. Resilience and psychosocial outcomes in parents of children with cancer. *Pediatr Blood Cancer.* 2014;61(3):552-557.
25. Yi JP, Vitaliano PP, Smith RE, Yi JC, Weinger K. The role of resilience on psychological adjustment and physical health in patients with diabetes. *Brit J of Health Psychol.* 2008;13:311-325.
26. Yi-Frazier JP, Cochrane K, Whitlock K, et al. Trajectories of acute diabetes-specific stress in adolescents with Type 1 Diabetes and their caregivers within the first year of diagnosis. *J Pediatr Psychol.* 2018;43:645-653.
27. Yi-Frazier JP, Smith RE, Vitaliano PP, et al. A person-focused analysis of resilience resources and coping in diabetes patients. *Stress and Health.* 2010;26(1):51-60.
28. Hilliard ME, Hagger V, Hendrieckx C, et al. Strengths, Risk Factors, and Resilient Outcomes in Adolescents With Type 1 Diabetes: Results From Diabetes MILES Youth-Australia. *Diabetes Care.* 2017;40:849-855.
29. Rosenberg AR, Yi-Frazier JP, Eaton L, et al. Promoting Resilience in Stress Management: A pilot study of a novel resilience-promoting intervention for adolescents and young adults with serious illness. *J Pediatr Psychol.* 2015;40:992-999.
30. Toprak D, McNamara S, Nay L, et al. Resilience in cystic fibrosis: a pilot feasibility study of the Promoting Resilience in Stress Management (PRISM) intervention (Abstract). *North American Cystic Fibrosis Conference.* 2017.
31. Rosenberg AR, Bradford M, McCauley E, et al. Promoting Resilience in Adolescents and Young Adults with Cancer: results from the PRISM randomized controlled trial (Original Research). *Cancer.* 2018;124:3909-3917.
32. Folkman S. Positive psychological states and coping with severe stress. *Soc Sci Med.* 1997;45(8):1207-1221.
33. Yi-Frazier JP, Hilliard M, Cochrane C, Hood KK. The impact of positive psychology on diabetes outcomes: a review. *Psychology* 2012;3:1116-1124.
34. Mulvaney SA, Rothman RL, Osborn CY, Lybarger C, Dietrich MS, Wallston KA. Self-management problem solving for adolescents with type 1 diabetes: intervention processes associated with an Internet program. *Patient Educ Couns.* 2011;85:140-142.
35. Andersen BL, Thornton LM, Shapiro CL, et al. Biobehavioral, immune, and health benefits following recurrence for psychological intervention participants. *Clin Cancer Res.* 2010;16(12):3270-3278.
36. Abualula NA, Jacobsen KH, Milligan RA, Rodan MF, Conn VS. Evaluating diabetes educational interventions with a skill development component in adolescents with Type 1 Diabetes: A systematic review focusing on quality of life. *Diabetes Educ.* 2016;42:515-528.
37. Grey M, Boland EA, Davidson M, Li J, Tamborlane WV. Coping skills training for youth with diabetes mellitus has long-lasting effects on metabolic control and quality of life. *J Pediatr* 2000;137(1):107-113.
38. Hilliard ME, Powell PW, Anderson BJ. Evidence-based behavioral interventions to promote diabetes management in children, adolescents, and families. *Am Psychol.* 2016;71:590-601.
39. Rosenberg AR, Yi-Frazier JP. Commentary: Resilience Defined: An Alternative Perspective. *Journal of Pediatric Psychology.* 2016;41:506-509.

40. Rosenberg AR, Syrjala KL, Martin P, et al. Resilience, health, and quality of life among long-term survivors of hematopoietic cell transplantation. *Cancer.* 2015;121:4250-4257.
41. Hilliard ME, Iturralde E, Weissberg Benchell J, Hood KK. The Diabetes Strengths and Resilience Measure for Adolescents With Type 1 Diabetes (DSTAR-Teen): Validation of a New, Brief Self-Report Measure. *J Pediatr Psychol.* 2017;42:995-1005.
42. Folkman S, Greer S. Promoting psychological well-being in the face of serious illness: when theory, research and practice inform each other. *Psychooncology.* 2000;9(1):11-19.
43. Burton NW, Pakenham KI, Brown WJ. Feasibility and effectiveness of psychosocial resilience training: a pilot study of the READY program. *Psychol Health Med.* 2010;15(3):266-277.
44. Folkman S, Greer S. Promoting psychological well-being in the face of serious illness: When theory, research and practice inform each other. *Psychooncology.* 2000;9:11-19.
45. Southwick SM, Charney DS. The science of resilience: implications for the prevention and treatment of depression. *Science.* 2012;338:79-82.
46. Karlson CW, Rapoff MA. Attrition in randomized controlled trials for pediatric chronic conditions. *J Pediatr Psychol.* 2009;34(7):782-793.
47. Weissberg-Benchell J, Rausch J, Iturralde E, Jedraszko A, Hood K. A randomized clinical trial aimed at preventing poor psychosocial and glycemic outcomes in teens with type 1 diabetes (T1D). *Contemp Clin Trials.* 2016;49:78-84.
48. Wysocki T, Harris MA, Greco P, et al. Randomized, controlled trial of behavior therapy for families of adolescents with insulin-dependent diabetes mellitus. *Journal of Pediatric Psychology.* 2000;25(1):23-33.
49. Kolko DJ, Perrin E. The integration of behavioral health interventions in children's health care: services, science, and suggestions. *J Clin Child Adolesc Psychol.* 2014;43:216-228.
50. Erickson SJ, Gerstle M, Feldstein SW. Brief interventions and motivational interviewing with children, adolescents, and their parents in pediatric health care settings: a review. *Arch Pediatr Adolesc Med.* 2005;159:1173-1180.
51. Agiostratidou G, Anhalt H, Ball D, et al. Standardizing clinically meaningful outcome measures beyond HbA1c for Type 1 Diabetes: a consensus report of the American Association of Clinical Endocrinologists, the American Association of Diabetes Educators, the American Diabetes Association, the Endocrine Society, JDRF International, The Leona M. and Harry B. Helmsley Charitable Trust, the Pediatric Endocrine Society, and the T1D Exchange. *Diabetes Care.* 2017;40:1622-1630.
52. Shapiro JB, Vesco AT, Weil LEG, Evans MA, Hood KK, Weissberg-Benchell J. Psychometric properties of the Problem Areas in Diabetes: Teen and Parent of Teen Versions. *J Pediatr Psychol.* 2017;epub ahead of print.
53. Nansel TR, Iannotti RJ, Liu A. Clinic-integrated behavioral intervention for families of youth with type 1 diabetes: randomized clinical trial. *Pediatrics.* 2012;124:e866-873.
54. Connor KM, Davidson JR. Development of a new resilience scale: the Connor-Davidson Resilience Scale (CD-RISC). *Depress Anxiety.* 2003;18(2):76-82.
55. Campbell-Sills L, Stein MB. Psychometric analysis and refinement of the Connor-davidson Resilience Scale (CD-RISC): Validation of a 10-item measure of resilience. *J Trauma Stress.* 2007;20(6):1019-1028.
56. Campbell-Sills L, Cohan SL, Stein MB. Relationship of resilience to personality, coping, and psychiatric symptoms in young adults. *Behav Res Ther.* 2006;44(4):585-599.
57. Phipps S, Long AM, Ogden J. Benefit Finding Scale for Children: preliminary findings from a childhood cancer population. *J Pediatr Psychol.* 2007;32(10):1264-1271.
58. Huston SA, Blount RL, Heidesch T, Southwood R. Resilience, emotion processing and emotion expression among youth with type 1 diabetes. *Pediatr Diabetes.* 2016;17(8):623-631.
59. Martin A, Rief W, Klaiberg A, Braehler E. Validity of the Brief Patient Health Questionnaire Mood Scale (PHQ-9) in the general population. *Gen Hosp Psychiatry.* 2006;28(1):71-77.

60. Kroenke K, Spitzer RL, Williams JBW. The PHQ-9 - Validity of a brief depression severity measure. *J Gen Intern Med.* 2001;16(9):606-613.
61. Lowe B, Grafe K, Kroenke K, et al. Predictors of psychiatric comorbidity in medical outpatients. *Psychosom Med.* 2003;65(5):764-770.
62. Lowe B, Unutzer J, Callahan CM, Perkins AJ, Kroenke K. Monitoring depression treatment outcomes with the patient health questionnaire-9. *Med Care.* 2004;42(12):1194-1201.
63. Hilliard M, Minard C, Marrero D, et al. Living with type 1 diabetes: A new approach to assessing diabetes-specific health-related quality of life in youth. . *Poster presentation at the Society of Pediatric Psychology Annual Conference.* 2019.
64. La Greca AM, Swales T, Klemp S, Madigan S. Self care behaviors among adolescents with diabetes (Abstract). *Ninth Annual Sessions of the Society of Behavioral Medicine*. Baltimore, MD: Society of Behavioral Medicine; 1988:A42.
65. Lewin AB, LaGreca AM, Geffken GR, et al. Validity and reliability of an adolescent and parent rating scale of type 1 diabetes adherence behaviors: the Self-Care Inventory (SCI). *J Pediatr Psychol.* 2009;34:999-1007.
66. Kichler JC, Kaugars AS, Maglio K, Alemzadeh R. Exploratory analysis of the relationships among different methods of assessing adherence and glycemic control in youth with type 1 diabetes mellitus. *Health Psychol.* 2012;31:35-42.
67. Datye KA, Patel NJ, Jaser SS. Measure of adherence and challenges in using glucometer data in youth with Type 1 Diabetes: rethinking the value of self-report. *J Diabetes Res.* 2017;epub ahead of print.
68. Markowitz JT, Laffel LMB, Volkening LK, et al. Validation of an abbreviated adherence measure for young people with Type 1 diabetes. *Diabetic Med.* 2011;28(9):1113-1117.
69. Iturralde E, Hood KK, Weissberg-Benchell J, Anderson BJ, Hilliard ME. Assessing strengths of children with type 1 diabetes: Validation of the Diabetes Strengths and Resilience (DSTAR) measure for ages 9 to 13. *Pediatr Diabetes.* 2019.
70. Hood KK, Butler DA, Anderson BJ, Laffel LM. Updated and revised Diabetes Family Conflict Scale. *Diabetes Care.* 2007;30(7):1764-1769.
71. Katz ML, Volkening LK, Dougher CE, Laffel LM. Validation of the Diabetes Family Impact Scale: a new measure of diabetes-specific family impact. *Diabet Med.* 2015;32:122701231.
72. Corathers SD, Yi-Frazier JP, Kichler JC, et al. Development and implementation of the Readiness Assessment of Emerging Adults with Type 1 Diabetes Diagnosed in Youth (READDY) Tool. *Diabetes Spectrum.* 2019;in press.
73. Halekoh U, Højsgaard S. A Kenward-Roger Approximation and Parametric Bootstrap Methods for Tests in Linear Mixed Models - The R Package pbkrtest. *Journal of Statistical Software.* 2014;58:1-30.
74. Benjamini Y, Hochberg Y. Controlling the false discovery rate: a practical and powerful approach to multiple testing. *Journal of the Royal Statistical Society.* 1995;57 (1):289–300.
75. Diehr P, Johnson LL. Accounting for missing data in end-of-life research. *J Palliat Med.* 2005;8 Suppl 1:S50-57.
76. Kurland BF, Heagerty PJ. Directly parameterized regression conditioning on being alive: analysis of longitudinal data truncated by deaths. *Biostatistics.* 2005;6(2):241-258.
77. Little R RD. *Statistical analysis with missing data.* New York: Wiley; 1987.
78. Rosenberg AR, Bona K, Wharton CM, et al. Adolescent and Young Adult Patient Engagement and Participation in Survey-Based Research: A Report From the "Resilience in Adolescents and Young Adults With Cancer" Study. *Pediatr Blood Cancer.* 2016;63(4):734-736.
79. Yi-Frazier JP, Fladeboe K, Klein V, et al. Promoting Resilience in Stress Management for Parents (PRISM-P): An intervention for caregivers of youth with serious illness. *Fam Syst Health.* 2017;35:341-351.
80. Hilliard ME, Weissberg-Benchell J, Hood KK. Psychometric properties of a diabetes resilience measure for adolescents [abstract]. *Diabetes* 2014;63:1244.

81. Mitchell S, Hilliard ME, Mednick L, Streisand R. Stress among fathers of young children with type 1 diabetes. *Families, Systems, and Health.* 2009;27:314-324.
82. Hilliard ME, Monaghan M, Cogen FR, Streisand R. Parent stress and child behaviour among young children with type 1 diabetes. *Child Care Health Dev.* 2011;37:224-232.
83. Hilliard ME, Guilfoyle SM, Dolan LM, Hood KK. Prediction of adolescents' glycemic control 1 year after diabetes-specific conflict: The mediating role of blood glucose monitoring adherence. *Archives of Pediatric and Adolescent Medicine.* 2011;165:624-629.
84. Hilliard ME, Herzer M, Dolan LM, Hood KK. Psychological screening in adolescents with type 1 diabetes predicts outcomes one year later. *Diabetes Res Clin Pract.* 2011;94:39-44.
85. Hilliard ME, Mann KA, Peugh JL, Hood KK. How poorer quality of life in adolescence predicts subsequent type 1 diabetes management and control. *Patient Educ Couns.* 2013;91:120-125.
86. Mackey ER, Hilliard ME, Berger SS, Streisand R, Chen R, Holmes C. Individual and family strengths: An examination of the relation to disease management and metabolic control in youth with type 1 diabetes. *Families, Systems, and Health.* 2011;29:314-236.
87. Hilliard ME, Wu YP, Rausch J, Dolan LM, Hood KK. Predictors of deteriorations in diabetes management and control in adolescents with type 1 diabetes. *J of Adolesc Health.* 2013;52:28-34.
88. Wu YP, Hilliard ME, Rausch J, Dolan LM, Hood KK. Family involvement with the diabetes regimen in young people: The role of adolescent depressive symptoms. *Diabet Med.* 2013;30:596-602.
89. Hilliard ME, Holmes CS, Chen R, Maher K, Robinson E, Streisand R. Disentangling the roles of parental monitoring and family conflict in adolescents' type 1 diabetes self-care. *Health Psychol.* 2013;32:388-396.
90. Anderson BJ, Brackett J, Ho J, Laffel LM. An office-based intervention to maintain parent-adolescent teamwork in diabetes management. Impact on parent involvement, family conflict, and subsequent glycemic control. *Diabetes Care.* 1999;22(5):713-721.
91. Anderson BJ, Wolf FM, Burkhart MT, Cornell RG, Bacon GE. Effects of peer-group intervention on metabolic control of adolescents with IDDM. Randomized outpatient study. *Diabetes Care.* 1989;12(3):179-183.
92. Anderson BJ, Laffel LM, Domenger C, et al. Factors Associated With Diabetes-Specific Health-Related Quality of Life in Youth With Type 1 Diabetes: The Global TEENs Study. *Diabetes Care.* 2017;40:1002-1009.
93. Katz ML, Volkening LK, Butler DA, Anderson BJ, Laffel LM. Family-based psychoeducation and care ambassador intervention to improve glycemic control in youth with type 1 diabetes: a randomized trial. *Pediatr Diabetes.* 2014;15:142-150.
94. Malik FS, Yi-Frazier JP, Taplin CE, et al. Improving the Care of Youth With Type 1 Diabetes With a Novel Medical-Legal Community Intervention: The Diabetes Community Care Ambassador Program. *Diabetes Educ.* 2018;44:168-177.
95. Pihoker C, Paris C, Imperatore G, et al. Age, Insulin regimen and HbA1C: The SEARCH for Diabetes in Youth Study. *Pediatric Diabetes.* 2007;8 (suppl. 7):11.
96. DeSalvo DJ, Ly T, Wadwa RP, et al. Continuous glucose sensor survival and accuracy over 14 consecutive days. *Diabetes Care.* 2016;39:e112-113.

---

[1] Include information if this protocol is associated with other IRB-approved studies (e.g. is this application the next part/phase of a previously approved application.

[2] In clinical trials, an endpoint is an event or outcome that can be measured objectively to determine whether the intervention being studied is beneficial. Some examples of endpoints are survival, improvements in quality of life, relief of symptoms, and disappearance of the tumor.

---

[3] Include information on a drug or biologic in this section if: (1) the study specifies the use of an approved drug or biologic; (2) the study uses an unapproved drug or biologic; (3) the study uses a food or dietary supplement to diagnose, cure, treat, or mitigate a disease or condition; or (4) data regarding subjects will be submitted to or held for inspection by the Food and Drug Administration (FDA). Only include information on a device in this section if: (1) the study evaluates the safety or effectiveness of a device; (2) the study uses a humanitarian use device (HUD) for research purposes; or (3) data regarding subjects will be submitted to or held for inspection by the FDA. Please note that mobile medical applications may meet the definition of a device – see FDA Guidance.

[4] See the Investigator Manual HRP-103 for sponsor requirements for FDA-regulated research.

[5] Explain what IND exemption category applies to the drug and why. Note that a drug is not exempt from an IND unless all criteria for one category are met. See “HRP-306: Drugs” for more information.

[6] Explain what IDE exemption category applies to the device and why. Note that a device is not exempt from an IDE unless all criteria for one category are met. See “HRP-307: Devices” for more information.

[7] Explain why the device is NOT a significant risk device. A significant risk device means an investigational device that: (a) is intended as an implant and presents a potential for serious risk to the health, safety, or welfare of a subject; (b) is purported or represented to be for use supporting or sustaining human life and presents a potential for serious risk to the health, safety, or welfare of a subject; (c) is for a use of substantial importance in diagnosing, curing, mitigating, or treating disease, or otherwise preventing impairment of human health and presents a potential for serious risk to the health, safety, or welfare of a subject; or (d) otherwise presents a potential for serious risk to the health, safety, or welfare of a subject.

[8] Be sure to indicate if controls will be included and include information about why control arms are ethically acceptable.

[9] Describe all of the research procedures being performed. Be sure to make it clear which procedures apply to each subject population. When applicable, describe how research procedures differ from standard of care and/or affect standard of care. Describe any audio/video recording that will be involved.

[10] Attach all surveys, scripts, and data collection forms to the “Supporting Documents” page.

[11] Include information about the frequency of data collection.

[12] See HRP-001 - SOP – Definitions for definition of banking. Type N/A if not applicable. If the data is subject to NIH Genomic Data Sharing Policies (e.g. you will submit data to dbGaP, NDAR, FITBIR), indicate here.

[13] If applicable, include a list of identifiers that will be banked.

[14] Be general (e.g., researchers’ lab, clinic, etc.)

[15] Generally, data and/or biospecimens should be released in a coded, non – identifiable manner.

[16] Include a description of the process used to verify and document that any required approvals have been obtained prior to release of data/biospecimens from the bank.

[17] You can allow for use for broad purposes

[18] This includes putting results and/or data in the subject medical records.

---

[19] If your population will differ from the representative population where the study will take place (e.g., race, ethnic group, or gender), provide a rationale for the differences.

[20] If you check a box below, be sure to include the additional safeguards associated with the population.

[21] Refer to HRP-416 CHECKLIST: Children.

[22] If the study is minimal risk, explain why. Must also include, as applicable: (1) why direct benefits are anticipated, (2) why risks are justified by anticipated benefit and/or the relationship between risk and prospective benefit compared to available alternatives, (3) why risk represents only minor increase over minimal risk, (4) how study procedures are reasonably commensurate with those inherent to the child's actual or expected conditions, (5) whether the interventions/procedures are likely to yield generalizable knowledge about the participant's condition and why it is of "vital importance" to understanding or amelioration of the participant's underlying disorder or condition, and (6) an explanation of what alternative methods/approaches were considered to make the above assessments (as applicable).

[23] This population may be wards of the state or any other agency, institution, or entity. Refer to HRP-416 CHECKLIST: Children, Section 6, for additional guidance on required considerations for this population.

[24] This refers to both cognitive impairments and adults who are incapacitated for any other reason. As applicable, refer to HRP-417 CHECKLIST: Cognitively Impaired Adults.

[25] Refer to HRP-413 CHECKLIST: Neonates and HRP-414 CHECKLIST: Neonates of Uncertain Viability.

[26] Refer to HRP-412 CHECKLIST: Pregnant Women.

[27] Refer to HRP-415 CHECKLIST: Prisoners

[28] Indicate how you will ensure that there is no coercion or undue influence

[29] A subject is considered "enrolled" when they consent to be in the study.

[30] Only applicable for multisite studies.

[31] i.e., numbers of subjects excluding screen failures.

[32] Payment for participation is not considered a benefit.

[33] For example, data will be double entered, data will be reviewed by another study team member to ensure accuracy, etc.

[34] If your study is multisite and there are differences in how confidentiality will be maintained by the coordination center and our local site, this should be explained in this section (e.g. local site will have samples that are linked to a person's name, but the coordination center will only receive coded samples without any links). Confidentiality regarding use of Social Media will be explained in a protocol section below.

[35] Applicable for studies that present more than minimal risk.

[36] Include information about who (describe in terms of role or group) will review the data.

[37] This should be specific to the social media you are using for the research.

---

[38] All communications that are directed towards subjects and specific to a particular study will require prior IRB review and approval. All non-IRB reviewable communications can be described in general terms by category – news stories, relevant publications – and representative examples of each can be provided.

[39] Applicable if the research involves more than minimal risk to subjects. If minimal risk, this section is N/A.

[40] If this is a multicenter study and subjects will be recruited by methods not under the control of the local site (e.g., call centers, national advertisements) those methods should also be described here.

[41] "Privacy interest" refers to a person's desire to place limits on whom they interact or whom they provide personal information.

[42] For example: medical records, CIS, clinical databases, other study records. If the study will access PHI for recruitment purposes without prior authorization from subjects, please address this in the HIPAA Authorization section below.

[43] Attach copies of these documents to the Recruitment Materials section of the study SmartForm. For printed advertisements, attach the final copy. For online advertisements, attach the final screen shots (including any images). When advertisements are taped for broadcast, send the final audio/video tape to IRB@seattlechildrens.org. You may attach the wording of the advertisement to the SmartForm prior to taping to preclude re-taping because of inappropriate wording, provided the IRB reviews the final audio/video tape.

[44]Include how you will ensure that subjects and/or their parent/legally authorized representative have sufficient opportunity to discuss and consider whether or not to participate in the research. .

[45] "Privacy interest" refers to a person's desire to place limits on whom they interact or whom they provide personal information.

[46] This section describes the way(s) in which the processes for this study will not follow Seattle Children's SOP.

[47] See HRP-090, HRP-091, and Investigator Manual HRP-103 for more information.

[48] Note the Short Form Consent may only be used when certain conditions are met. See HRP-091 for requirements for Short Form consent form use.

[49] Seattle Children's IRB prohibits the exclusion of non-English speaking populations from research unless there is sufficient justification for the exclusion. See Investigator Manual HRP-103 for more information.

[50] For research conducted in the state, review "SOP: Legally Authorized Representatives, Children, and Guardians (HRP-013)" to be aware of which individuals in the state meet the definition of "children." The age of majority in Washington is 18; however, sometimes younger children have ability to consent for certain types of care (e.g. sexual reproduction/health; mental health; drug/alcohol treatment). For research conducted outside of the state, provide information that describes which persons have not attained the legal age for consent to treatments or procedures involved the research, under the applicable law of the jurisdiction in which research will be conducted. One method of obtaining this information is to have a legal counsel or authority review your protocol along the definition of "children" in "SOP: Legally Authorized Representatives, Children, and Guardians (HRP-013)." If the sites in other states in the study are conducting their own IRB review, you do not need to worry about this--type N/A. If you are conducting research and are actively recruiting participants outside of Washington who are NOT coming to SCH to give consent and who will be covered under SCH IRB approval, this section should be addressed in your protocol.

[51] For minimal risk studies and greater than minimal risk studies that offer a prospect of benefit, the IRB generally requires one parent to provide permission for the child to participate.

---

[52] If parental permission will not be obtained, please address this in the Waiver or Alteration of Consent Process below.

[53] See HRP-013 for more information.

[54] The IRB generally follows the following guidelines for written assent: children 7-12 should provide written assent on the "simple" assent form (HRP-502G); children 13-17 should provide written assent by co-signing the parental permission form (HRP-502A). The IRB will consider other assent scenarios (e.g. verbal assent for some or all children; not requiring assent for some or all children; or waiving assent): please provide details about the plan for your study. See HRP-090 and HRP-416 for more information on waiving assent and when assent is not necessary.

[55] See Appendix A-13 of the Investigator Manual HRP-103 for requirements for re-consent at age 18. If you think you meet the conditions for a waiver at 18, please address this in the Waiver or Alteration of Consent Process below.

[56] See "HRP-417 Cognitively Impaired Adults" for further information.

[57] For example: durable power of attorney for health care, court appointed guardian for health care decisions, spouse, and adult child. If you are following HRP-013 in order to make this determination, simply state that in this section. For research conducted in the state, review "SOP: Legally Authorized Representatives, Children, and Guardians (HRP-013)" to be aware of which individuals in the state meet the definition of "legally authorized representative." For research conducted outside of the state, provide information that describes which individuals are authorized under applicable law to consent on behalf of a prospective subject to their participation in the procedure(s) involved in this research. One method of obtaining this information is to have a legal counsel or authority review your protocol along the definition of "legally authorized representative" in "SOP: Legally Authorized Representatives, Children, and Guardians (HRP-013)." If the sites in other states in the study are conducting their own IRB review, you do not need to worry about this--type N/A. If you are conducting research and are actively recruiting participants outside of Washington who are NOT coming to Washington to give consent and who will be covered under SCH IRB approval, this section should be addressed in your protocol.

[58] The IRB may allow the person obtaining assent to document assent on the consent document.

[59] For example: consent/parental permission will not be obtained, required information will not be disclosed, the research involves deception, waiver for participants who turn 18, waiver for information collected about a non-present parent, or other waivers as necessary.

[60] The IRB needs to make all the waiver findings and key to this determination is that the IRB understand why it is not practicable to do the research without a waiver of consent. You need to provide a rationale in order for the IRB to consider whether the waiver criteria are met. See "HRP-410: Waiver or Alteration of the Consent Process" for further information.

[61] Possible reasons might include: a) you are not collecting information that could put subjects or their families at harm, e.g., affect eligibility for insurance, employability, stigmatization; b) you are not collecting information that would alter or affect the subject's care; c) any publication or presentation of research results would be done in a manner that would never reveal an individual's identity either directly or indirectly.

[62] Possible reasons could be: a) inability to locate families because of the lengthy time period over which the records/samples were created; b) many of the subjects whose records, data, or biospecimens will be used may have died and contacting the families about the research could cause harm and anguish to families; c) all eligible patients must be included in the study for the results to be meaningful.

[63] See "HRP 419: Waiver of Consent for Emergency Research" for further information.

[64] This section describes the ways in which the procedures will not be following Seattle Children's SOP.

[65] See "HRP-411: Waiver or Written Documentation of Informed Consent" for further information.

[66] An information sheet template can be found in the Click IRB Library and should be attached to the consent form of the study SmartForm. For internet research, the information sheet can be translated to an on-line format, if desired.

[67] The IRB sometimes requires a script if you are having the consent conversation over the phone rather than in person. Templates for a consent script are available on the IRB website on the Participant Recruitment page and should be attached to the study SmartForm.

[68] PHI is health information that is also identifiable because it includes one or more of the 18 HIPAA identifiers. See Investigator Manual HRP-103 for the list of HIPAA identifiers.

[69] If your study involves using or creating PHI and your only contact with participants is online, you can request an alteration of HIPAA authorization to remove the signature requirement. As an alternative to a waiver of documentation of consent and an alteration of HIPAA authorization, you must demonstrate that the electronic consent signatures are compliant with applicable state/international law (in Washington, see RCW 19.34.300).

[70] For example: altering HIPAA elements for international research.

[71] Possible reason could be: the nature of the research is specific to individuals' health and requires access to individuals' health records.

[72] See "HRP-316: Payments" for further information.

[73] Methods of payment include check, ClinCard, gift cards, etc. Provide details on who will be the recipient of the payment (parent or child).

[74] Reimbursement is used when the subject is paid back for travel expenses such as transportation, food, childcare, or lodging. Reimbursement is generally distributed to person who incurred cost (usually parent) and requires receipts to be submitted.

[75] This could include things like fuel/transportation costs, parking, and/or childcare.

[76] Type N/A if this section does not apply.

[77] Provide enough information to convince the IRB that the principal and/or co-investigator(s) are appropriately qualified to conduct and supervise the proposed research. When applicable, describe their prior clinical experience with the test article or study-related procedures, or describe their knowledge of the local study sites, culture, and society.

[78] For example, as appropriate: (1) Justify the feasibility of recruiting the required number of suitable subjects within the agreed recruitment period. For example, how many potential subjects do you have access to? What percentage of those potential subjects do you need to recruit? (2) Describe the time that you will devote to conducting and completing the research. (3) Describe the facilities in which the research will be conducted. (4) Describe the availability of medical or psychological resources that subjects might need as a result of an anticipated consequences of the human research. (5) Describe your process to ensure that all persons assisting with the research are adequately informed about the protocol, the research procedures, and their duties and functions.

[79] Including communication between sites of current study document versions and modifications.

[80] If you check the box, you are required to conduct your study according to the principles outlined at https://www.ich.org/products/guidelines/efficacy/efficacy-single/article/integrated-addendum-good-clinical-practice.html.
