# Supplementary material for: Promoting Resilience in Stress Management for Adolescents With Type 1 Diabetes: A Randomized Clinical Trial
Source: JAMA Netw Open. 2024 Aug 19;7(8):e2428287. doi: 10.1001/jamanetworkopen.2024.28287 (PMC11333977; doi:10.1001/jamanetworkopen.2024.28287)
Supplement: Supplement 2. — eTable 1. Unadjusted Results for Outcomes of PRISM vs Usual Care Over the 12 Study Months eTable 2. Effect Sizes on Outcomes at 6 and 12 Months eTable 3. Adjusted Regression Analysis With Multiple Imputations (MI) [file jamanetwopen-e2428287-s002.pdf]

Supplementary Online Content

Yi-Frazier JP, Hilliard ME, O'Donnell MB, et al. Promoting Resilience in Stress Management for adolescents with type 1 diabetes. *JAMA Netw Open*. 2024;7(8):e2428287. doi:10.1001/jamanetworkopen.2024.28287

eTable 1. Unadjusted Results for Outcomes of PRISM vs Usual Care Over the 12 Study Months
eTable 2. Effect Sizes on Outcomes at 6 and 12 Months
eTable 3. Adjusted Regression Analysis With Multiple Imputations (MI)

This supplementary material has been provided by the authors to give readers additional information about their work.

© 2024 Yi-Frazier JP et al. *JAMA Network Open*.

eTable 1. Unadjusted Results for Outcomes of PRISM vs Usual Care Over the 12 Study Months

	Baseline Mean (SD)	6-Month Mean (SD)	Δ1[a] Mean (SD)	P-value	12-Month Mean (SD)	Δ2[b] Mean (SD)	P-value
**Primary outcomes:**							
HbA1c							
Usual Care	8.6(2.2)	8.6(1.8)	0.3 (1.0)		8.3(1.6)	0.1 (1.3)	
PRISM	8.8(1.9)	8.7(1.8)	0.0(1.5)	0.2	8.5(1.8)	-0.3 (1.5)	0.09
Diabetes Distress (PAID[c])							
Usual Care	47.3(13.0)	39.9(15.0)	-7.3 (14.0)		38.9(16.0)	-7.7 (-14.7)	
PRISM	46.1(11.5)	36.2(12.5)	-9.8 (12.2)	0.2	33.5(13.1)	-12.4 (-13.2)	0.05
**Secondary outcomes:**							
Resilience (CD-RISC)							
Usual Care	24.5(6.4)	25.7(7.3)	1.3 (5.9)		26.3(8.0)	1.9 (6.1)	
PRISM	24.3(6.0)	25.2(6.8)	0.8 (5.5)	0.6	26.0(7.8)	1.8 (6.4)	0.09
Diabetes Resilience (DSTAR)							
Usual Care	43.8(6.9)	45.0(7.7)	0.8 (6.1)		45.9(7.1)	1.1 (6.5)	
PRISM	45.0(5.8)	46.9(6.7)	1.9 (6.2)	0.2	48.4(7.3)	3.0 (6.8)	0.08
Self-Management Behaviors (DSMQ)							
Usual Care	20.2 (6.7)	17.9 (9.1)	-2.3 (9.1)		16.6 (10.0)	-3.6 (9.6)	
PRISM	19.8 (5.9)	19.9 (7.0)	0.1 (7.8)	0.07	19.6 (8.0)	-0.2 (7.9)	**0.01**
Health-Related Quality of Life (T1DAL)							
Usual Care	49.2(13.7)	53.5(15.0)	3.1 (11.5)		54.2(16.0)	4.0 (11.4)	
PRISM	51.7(14.5)	56.9(15.4)	3.6 (10.8)	0.8	60.4(15.9)	7.6 (13.3)	0.10

[a]Δ1: Within-subject change between 6-month and baseline
[b]Δ2: Within-subject change between 12-month and baseline
[c] PAID (Problem Areas in Diabetes-Teen Version), CD-RISC (Connor-Davidson Resilience Scale), DSTAR (The Diabetes Strengths and Resilience Measure), DSMQ (Diabetes Self-Management Questionnaire), T1DAL (Type 1 Diabetes and Life Questionnaire)

© 2024 Yi-Frazier JP et al. *JAMA Network Open*.

eTable 2. Effect Sizes on Outcomes at 6 and 12 Months

**Outcomes**	**Cohen's d (95% CI)**	
	**6-Months**	**12-Months**
HbA1c	0.08 (-0.24, 0.39)	0.11 (-0.23, 0.44)
Diabetes Distress (PAID[a])	-0.27 (-0.59, 0.05)	**-0.37 (-0.7, -0.05)**
Resilience (CD-RISC)	-0.07 (-0.39, 0.25)	-0.03 (-0.36, 0.29)
Diabetes Resilience (DSTAR)	0.26 (-0.06, 0.58)	**0.35 (0.03, 0.68)**
Self-Management Behavior (DSMQ)	0.24 (-0.06, 0.54)	**0.33 (0.03, 0.63)**
Health-Related Quality of Life (T1DAL)	0.23 (-0.1, 0.55)	**0.39 (0.06, 0.72)**

[a]PAID (Problem Areas in Diabetes-Teen Version), CD-RISC (Connor-Davidson Resilience Scale), DSTAR (The Diabetes Strengths and Resilience Measure), DSMQ (Diabetes Self-Management Questionnaire), T1DAL (Type 1 Diabetes and Life Questionnaire)

© 2024 Yi-Frazier JP et al. *JAMA Network Open*.

eTable 3. Adjusted Regression Analysis With Multiple Imputations (MI)

Outcomes	Adjusted difference in changes from baseline PRISM vs. usual care[a]			
	6-Months		12-Months	
	β (95% CI)[b]	P-value[c]	β (95% CI)	P-value
HbA1c	-0.2 (-0.6, 0.2)	0.3	-0.3 (-0.7, 0.2)	0.3
Diabetes Distress (PAID)	-2.7 (-6.3, 0.9)	0.1	-4.6 (-8.2, -0.9)	**0.01**
Resilience (CD-RISC)	-0.4 (-2.2, 1.5)	0.7	-0.1 (-1.9, 1.7)	>0.9
Diabetes Resilience (DSTAR)	1.0 (-1.0, 2.9)	0.3	1.8 (-0.1, 3.8)	0.07
Self-Management Behaviors (DSMQ)	2.4 (-0.1, 5.0)	0.06	3.4 (0.9, 5.9)	**0.009**
Health-Related Quality of Life (T1DAL)	0.6 (-3.0, 4.3)	0.7	3.5 (-0.1, 7.2)	0.06

[a] Based on linear mixed effects regression models with the study group main effect, time main effect, group-by-time interaction. The stratification factor study site was controlled for as covariate.

[b] β's corresponded to the group-by-time interactions in the regression models

[c] Based on F-test with Kenward-Roger approximation to degrees of freedom

[d] PAID (Problem Areas in Diabetes-Teen Version), CD-RISC (Connor-Davidson Resilience Scale), DSTAR (The Diabetes Strengths and Resilience Measure), DSMQ (Diabetes Self-Management Questionnaire), T1DAL (Type 1 Diabetes and Life Questionnaire)

© 2024 Yi-Frazier JP et al. *JAMA Network Open*.
