# Supplementary material for: Promoting Resilience in Stress Management for Adolescents With Type 1 Diabetes: A Randomized Clinical Trial
Source: JAMA Netw Open. 2024 Aug 19;7(8):e2428287. doi: 10.1001/jamanetworkopen.2024.28287 (PMC11333977; doi:10.1001/jamanetworkopen.2024.28287)
Supplement: Supplement 3. — Data Sharing Statement [file jamanetwopen-e2428287-s003.pdf]

Data Sharing Statement

Yi-Frazier. Promoting Resilience in Stress Management for Adolescents With Type 1 Diabetes. *JAMA Netw Open*. Published August 19, 2024. doi:10.1001/jamanetworkopen.2024.28287

Data
Data available: No

Additional Information
Explanation for why data not available: Data will be available based on pre-approved IRB stipulations for minor-aged patients
